# Management of adverse events in patients with pediatric low-grade glioma treated with MAPK-directed therapies: Delphi consensus recommendations and unmet needs

**DOI:** 10.1093/nop/npag006

**Published:** 2026-02-06

**Authors:** Darren Hargrave, Daniel C Bowers, Stewart Goldman, Grant T Liu, Jennifer T Huang, Hanneke M van Santen, Nathan J Robison, Michal Zapotocky, Eric Bouffet

**Affiliations:** Great Ormond Street Hospital for Children, London, UK; UCL Great Ormond Street Institute of Child Health, London, UK; Simmons Comprehensive Cancer Center and the Department of Pediatrics, University of Texas Southwestern Medical Center, Dallas, Texas, USA; Department of Child Health, University of Arizona College of Medicine- Phoenix, Phoenix Children’s Hospital, Phoenix, Arizona, USA; Division of Ophthalmology, Children’s Hospital of Philadelphia, Philadelphia, Pennsylvania, USA; Dermatology Section, Boston Children’s Hospital, Boston, Massachusetts, USA; Princess Máxima Center for Pediatric Oncology and Department of Pediatric Endocrinology, Wilhelmina Children’s Hospital, UMC, Utrecht, The Netherlands; Cancer and Blood Disease Institute, Children’s Hospital Los Angeles, Los Angeles, California, USA; Department of Paediatric Haematology and Oncology, Second Faculty of Medicine, Charles University, Prague, Czech Republic; Center for Pediatric Neuro-oncology, Motol University Hospital, Prague, Czech Republic; Division of Hematology/Oncology, University of Toronto, Hospital for Sick Children (SickKids), Toronto, Ontario, Canada

**Keywords:** adverse events, BRAF inhibitor, MEK inhibitor, pediatric low-grade glioma, RAF inhibitor

## Abstract

**Background:**

Pediatric low-grade gliomas (pLGGs) are the most common childhood central nervous system (CNS) tumors. Targeted therapies are effective treatments in patients with pLGGs harboring mutations in the mitogen-activated protein kinase (MAPK)/extracellular signal-regulated kinase 1/2 (ERK) signaling pathway. Understanding the toxicity profile and tolerability of emerging MAPK inhibitors (MAPKi) and how adverse events (AEs) can be managed to avoid treatment discontinuation, interruption, or dose reduction is important for optimizing clinical benefit.

**Methods:**

A modified Delphi consensus initiative was conducted to provide recommendations on the monitoring and management of AEs that occur with MAPKi in patients with pLGG. A 9-member steering committee was convened to develop statements based on the findings of a comprehensive literature review of AEs reported with the use of MAPKi in pediatric cancers. Consensus on statements was determined via online surveys completed by a large, global panel of experts in pLGG.

**Results:**

Of the 129 statements drafted, consensus (≥75% agreement) among 82 global experts in pLGG was reached for 50 statements, mostly pertaining to the general management of AEs occurring with MAPKi and the management of cutaneous AEs. Consensus statements include guidance on skin care and specific cutaneous conditions. Many AEs were rare with limited evidence or experience to achieve consensus recommendations.

**Conclusions:**

The consensus statements developed provide guidance and recommendations for the management of common AEs in patients with pLGG treated with MAPKi. Sharing this knowledge may lead to patients with pLGG achieving optimal benefit from MAPKi while minimizing and effectively managing AEs.

Key Points

- MAPK inhibitors (MAPKi) are emerging treatments for pediatric low-grade glioma.
- The most common and challenging AEs that occur with MAPKi therapy are cutaneous.
- Expert consensus was reached on recommendations to manage common cutaneous AEs.

Importance of the Study

In this modified Delphi consensus initiative, experts from across the globe came together to develop recommendations for the management of adverse events (AEs) in patients with pediatric low-grade gliomas (pLGGs) being treated with MAPK pathway-directed therapies. This is the first study of its kind to provide best practice guidance on management of AEs for these emerging therapies in this patient population. The initiative also highlighted areas where additional data and clinical experience are needed to understand how best to manage less common AEs, so that the clinical benefit of MAPK inhibitors can be optimized in pLGG.

Low-grade gliomas (LGGs) are the most common central nervous system (CNS) tumors that occur in childhood.^1^ Goals of treatment for pediatric LGGs (pLGGs) include removing, regressing, or stabilizing the tumor and minimizing any functional effects, for example, on vision, motor, and cognitive function, or long-term morbidity to ensure optimal quality of life for the patient.^1^ Although many cases of pLGG can be managed with surgical resection and have a good clinical outcome, complete resection is not always possible as tumors may arise in areas of the CNS that are not suitable for resection due to the high risk of morbidity.^1^ Adjuvant therapies, including chemotherapy and radiation therapy, are required for these situations, but many times these tumors become a chronic condition that requires multiple courses of therapies.^1^^,^^2^ Chemotherapy has resulted in 5-year progression-free survival (PFS) rates of approximately 45%-55%; however, more than half of patients with pLGG will require additional treatment to achieve disease control.^1^^,^^2^

The majority of pLGGs harbor mutations in the MAPK/ERK signaling pathway,^3^ which is key for cell proliferation and survival. Therapies that target specific alterations in MAPK pathway genes, for example, *BRAF* V600E, have been developed to treat a variety of solid tumors and include type I BRAF, type II RAF, and MEK inhibitors.^1^

In a phase 2, randomized, clinical study, the combination of the BRAF inhibitor (BRAFi), dabrafenib, and the MEK inhibitor (MEKi), trametinib, was compared with standard chemotherapy (vincristine and carboplatin) in patients with pLGG with BRAF V600 mutations. The combination of dabrafenib and trametinib resulted in an overall response rate (ORR) of 47% versus 11% with chemotherapy and PFS of 20.1 months versus 7.4 months with chemotherapy.^4^ As a result, the combination of dabrafenib and trametinib was the first treatment approved in pLGG with BRAF V600 mutations in patients aged 1 year and above requiring systemic therapy.^5^ More recently, in an interim analysis of an ongoing phase 2 study, the type II RAF inhibitor (RAFi), tovorafenib, was shown to result in an ORR of 51%, and a median duration of response of 13.8 months in patients with relapsed/refractory *BRAF*-altered pLGG.^6^ Patients with known or suspected neurofibromatosis type 1 were excluded from the tovorafenib study.^6^ These data supported the accelerated approval of tovorafenib for use in patients aged 6 months and older with relapsed or refractory pLGG harboring a *BRAF* fusion or rearrangement, or BRAF V600 mutation.^7^ Other BRAFi and MEKi have been used to treat pLGG in early phase clinical studies^8–12^ or off label.

Although the optimal treatment duration has not been established, targeted therapies, at least in clinical trials, have typically been administered for 2 years or longer.^1^ Rapid tumor regrowth has been reported when targeted therapy is discontinued,^13^ particularly in patients with BRAF V600-mutated pLGG;^14^ however, pLGGs often respond when rechallenged with targeted therapy.^13^ In BRAF V600-mutated pLGGs, rapid progression occurred in more than 75% of cases at a median of 2.3 months when treatment with BRAF inhibition was discontinued, but an objective response was achieved in 90% of patients when treatment was resumed.^14^ Consequently, patients with pLGG often require long-term targeted treatment.^1^ As such, it is important to understand the toxicity profile and tolerability of these MAPK inhibitor (MAPKi) treatments and how any adverse events (AEs) can be managed to avoid dose reduction, treatment interruption, or discontinuation. Rash (eczematous and acneiform), paronychia, gastrointestinal AEs (nausea, vomiting, and diarrhea), decreased cardiac function, creatinine phosphokinase (CPK) elevations, retinal toxicity, weight gain, decreased growth velocity, and fatigue have all been observed with MAPKi therapies with varying frequencies.^15^

There is currently an unmet need for guidance as to how to manage AEs with these emerging MAPKi therapies in pLGG. Here, we gathered a panel of global healthcare professionals (HCPs) with clinical experience with MAPKis in patients with pLGG in a modified Delphi exercise to support and guide clinicians around the world in monitoring and managing treatment-emergent AEs (TEAEs) arising from the use of MAPKis, with the overall goal of preserving quality of life and reducing the treatment burden associated with pLGG.

## Materials and Methods

**Figure 1.**
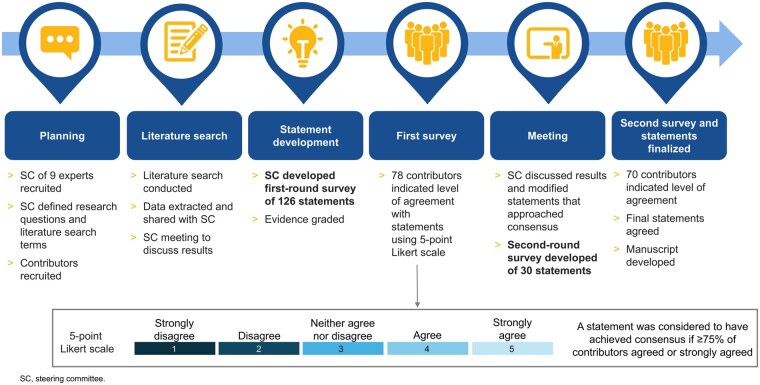
Modified Delphi process.

An overview of the modified Delphi process used is provided in Figure 1; the study protocol was not prospectively registered. To form the steering committee, a multidisciplinary panel of 9 HCPs from 5 countries was convened, consisting of 6 pediatric neuro-oncologists, a dermatologist, a neuro-ophthalmologist, and an endocrinologist; D.H. and E.B. were co-chairs. The steering committee met to agree on research questions, search strings, keywords, and inclusion/exclusion criteria for a literature search performed to identify publications containing information on AEs occurring with the use of MAPKis in pediatric cancers, see Supplementary Methods and Tables S1 to S6 for details.

The MAPKis included in the literature searches were type I BRAFi (dabrafenib, encorafenib, vemurafenib), type II RAFi (tovorafenib), and MEKi (cobimetinib, binimetinib, trametinib, mirdametinib, pimasertib, selumetinib). Articles were limited to those that included pediatric, adolescent, and young adult patients (<25 years of age). PubMed (including MEDLINE), Web of Science (Core Collection and BIOS), and Cochrane Trials and Reviews were searched between January 1, 2014, and April 22, 2024 (Tables S1-S4). Congresses held between January 2018 and May 2024 were also searched for keywords (Table S5).

**Figure 2.**
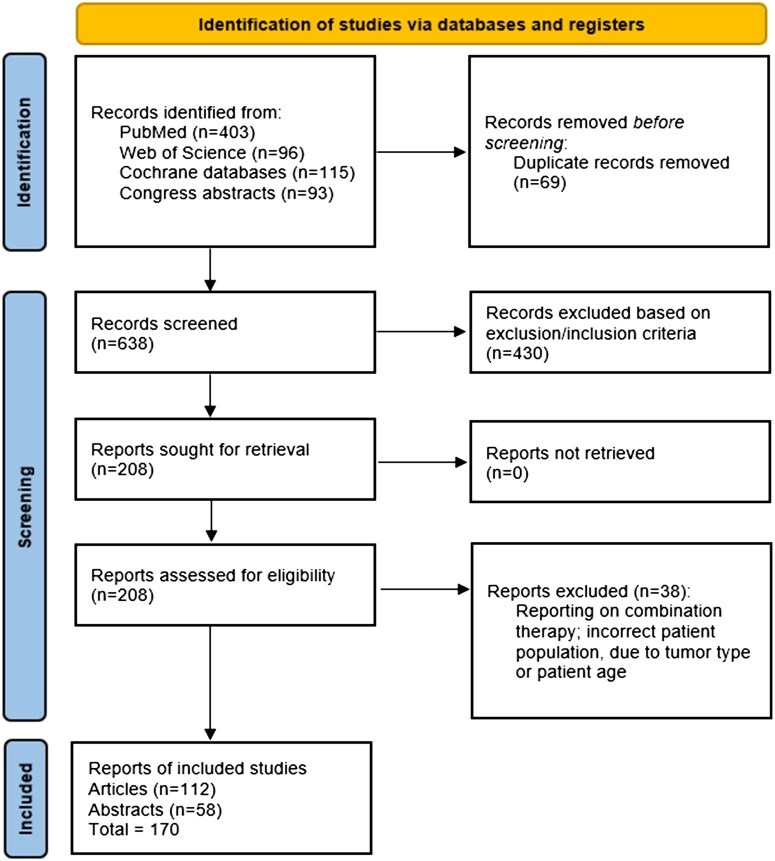
Flow diagram for literature search.

The literature search identified 638 articles and abstracts, of which 208 were selected for data extraction after screening; 112 articles and 58 abstracts contained information relevant for the development of statements (Figure 2). Articles identified were graded according to their level of evidence using a modified version of the Jovell/Navarro-Rubi grading criteria (Table S7). All articles and abstracts identified for the development of statements, and their level of evidence, are provided in Table S8.

The findings of the literature search facilitated the development of statements relating to: (1) incidence and severity of AEs; (2) incidence and severity of AEs leading to dose modification, discontinuation, or death; (3) risk factors for the development of AEs; (4) management of AEs; and (5) patient-reported outcomes and quality of life related to AEs. The 2 co-chairs and the steering committee met to agree on the first-round survey statements, which were then circulated to contributors invited to participate in surveys to establish consensus on draft statements.

The contributors were nominated by the steering committee or were authors on relevant publications in the field. Contributors were also asked to nominate colleagues working in the following specialties: cardiology, dermatology, endocrinology, ophthalmology, and nursing, who had experience treating pediatric patients with MAPKis. Surveys were completed via an online portal, with contributors selecting their level of agreement with draft statements using a 5-point Likert scale (strongly agree, agree, neither agree or disagree, disagree, strongly disagree). A free text response box was also provided for feedback to help guide modification of statements that did not achieve consensus.

Statements were considered to have achieved consensus if ≥75% of contributors strongly agreed or agreed with the statement; statements that approached consensus (65%-<75%) in the first-round survey (conducted January 28 to February 12, 2025) were discussed further among the steering committee and most were modified and resurveyed in the second-round survey (conducted March 3-March 13, 2025); any statements that did not achieve consensus (<65%) were discarded.

Contributors were not incentivized to complete the surveys, and the steering committee did not participate in the surveys. All survey responses were anonymized before analysis.

## Results

Based on the identified literature, level of evidence, and expert opinion, 126 statements were drafted and agreed upon by the steering committee. The first-round survey was sent to 92 experts, of which 78 contributors (response rate: 85%) completed the survey. Of the 126 statements included in the first-round survey, consensus was achieved for 31 statements (25%) and was approached (65%-<75% of contributors in agreement) for 27 statements (21%). No consensus was achieved for the remaining 68 statements (54%). The 27 statements that approached consensus were reviewed and revised by the steering committee into 30 statements for the second-round survey, which was completed by 70/92 contributors (response rate: 76%). In the second-round survey, consensus was achieved for 19 of the 30 statements. Overall, consensus was reached for 50 of 129 (39%) statements. Statements that did not achieve consensus (n = 79, 61%) are detailed in Table S9.

**Figure 3.**
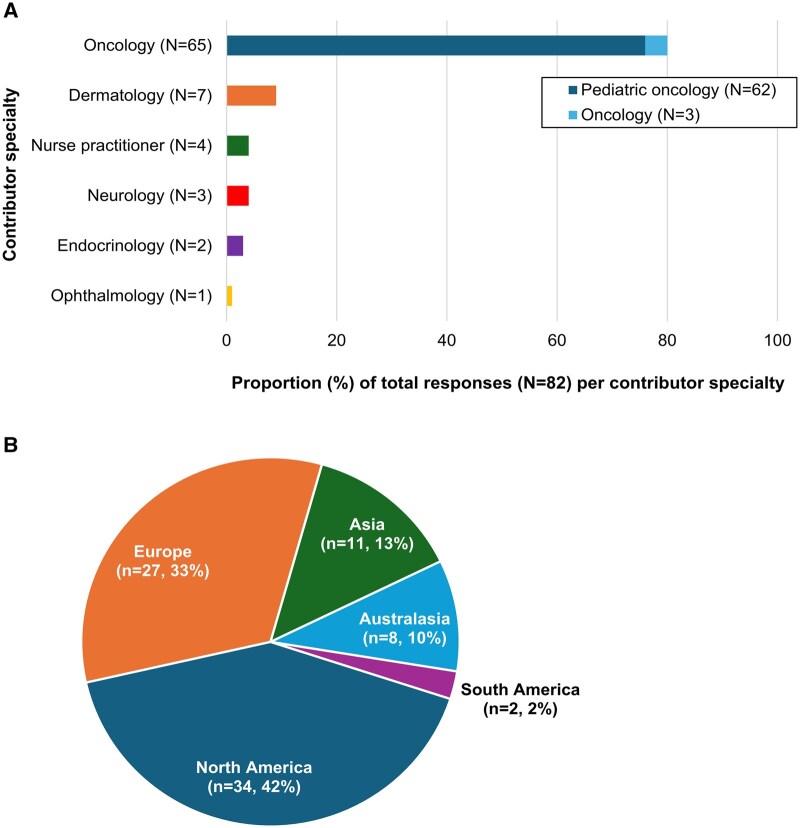
Overview of expert contributors. Number of experts contributing to Delphi surveys by specialty (A), and geographic location (B).

Across both surveys, 82 contributors based in 29 countries participated. Their specialties and geographic location are shown in Figure 3. Most experts (*n* = 55, 67%) were from centers in the United States and Europe. Sixty-five of the experts were oncologists, of which 62 were pediatric oncologists. Seven were dermatologists and the remaining 10 experts were nurse practitioners (*n* = 4), neurologists (*n* = 3), endocrinologists (*n* = 2), and ophthalmologists (*n* = 1).

The following sections reflect the recommendations of the expert panel based on the consensus statements for each of the selected AEs.

### Incidence and General Management of AEs

The statements were developed based on available evidence for AEs occurring with MAPKi therapies in pediatric cancers from phase 1 and 2 trials and observational studies; however, a large proportion of the evidence was from retrospective studies and case reports, and long-term data are limited. Statements reaching consensus for incidence and general management of AEs are shown in Table 1.

**Table 1.** Expert consensus on incidence and general management of AEs in pediatric patients treated with MAPKi therapies.

Consensus statement		Level of consensus (%)
1	MAPKi therapies, including type I BRAFi (vemurafenib, dabrafenib), the type II RAFi tovorafenib, and MEKi (trametinib, selumetinib), share common AEs due to their inhibition of the MAPK/ERK pathway, affecting both tumor and healthy cells	85
2	Life-threatening AEs associated with MAPKi therapies in pediatric patients are rare	89
3	Common AEs across all MAPKi therapies include skin toxicities, gastrointestinal side effects, endocrine effects, fatigue, and fever	87
4	There is a good understanding of most MAPKi-associated AEs to allow recommendations for therapy	79
5	Management strategies for AEs are based upon published literature and medical provider’s experience with MAPKi therapies	87
6	More information is needed regarding monitoring and treatment of long-term AEs	96
7	AEs associated with MAPKi therapies do overlap, but mechanisms and toxicities differ. Consult specific guidelines for each agent’s unique AEs	80
8	Almost two-thirds of publications (∼60%) involving MAPKi therapies in pediatric populations are in either pLGG or plexiform neurofibromas	78
9	Conclusions as to the frequency of treatment-associated AEs may be transferable across histologies to other treated tumor types, but age, comorbidities, and other treatments should also be considered	96
10	MAPKi therapies should only be prescribed, and their management overseen, by an appropriate oncology team in a multidisciplinary setting	87
11	Ensuring appropriate education of patients and caregivers on the potential AEs is essential to improve patient outcomes, manage patient/caregiver expectations, and aid treatment adherence	95
12	Additionally, patients/caregivers should be educated on the need for proactive AE management in order to control some AEs, particularly cutaneous toxicities	94
13	In the presence of AEs, periodical assessment of the need for MAPKi therapy and whether a guided (multidisciplinary team) break from therapy should be considered	90
14	MAPKi therapy can be re-initiated on a trial basis, after a break in treatment, to assess if the patient can cope better with the benefit or risk of therapy	87
15	It is unknown whether MAPKi therapy in childhood and adolescence may be associated with late-emerging health sequelae in adulthood; vigilance is therefore required in long-term follow-up	81
16	Further research is required into late-emergent AEs after MAPKi therapy, as well as AEs associated with long-term MAPKi therapy	81

Abbreviations: AEs, adverse events; BRAFi, BRAF inhibitor; MAPKi, MAPK inhibitor; MEKi, MEK inhibitor; pLGG, pediatric low-grade glioma; RAFi, RAF inhibitor.

Due to their shared role in inhibiting the MAPK/ERK pathway, MAPKi therapies (type I BRAFi, type II RAFi, and MEKi) have AEs in common; these include cutaneous toxicities, gastrointestinal AEs, endocrine AEs, fatigue, and fever. Fortunately, AEs occurring in response to MAPKi therapies are rarely life-threatening. The safety of MAPKi therapies has mostly been studied in pLGGs or plexiform neurofibromas; however, there was consensus that the frequency of treatment-related AEs with MAPKis may be transferable to other treated tumor types.

Overall, there was consensus that there is sufficient data and understanding among specialists to provide recommendations on managing common AEs in pediatric patients, although this may in part be based on experience of using these treatments in adults and more information on monitoring and treating long-term AEs in children and adolescents is needed. There are, however, differences in terms of mechanism and toxicities between the MAPKi drug classes that clinicians need to be aware of, and in these cases guidance for drug-specific AEs should be followed. Clinicians should follow local guidelines and determine availability and accessibility when choosing a particular treatment for their patients, and use should reflect the severity of the AEs observed.

Having a multidisciplinary team involved in patient care is recommended, but with MAPKi therapies being prescribed and monitored by oncology. This approach should ensure that any AEs that arise can be effectively managed; this may include MAPKi treatment interruption, if deemed appropriate by the multidisciplinary team. Educating caregivers and patients on potential AEs and how they can be proactively managed is also important to set their expectations and support treatment adherence to these chronic therapies; education on skin care from dermatologists was noted to be of particular importance.

It was agreed that the long-term impact of MAPKi therapy in childhood and adolescence is unknown; further research and long-term follow-up are needed to determine whether late-emerging health sequelae occur in adulthood and the impact of long-term MAPKi treatment on AEs.

### Cutaneous AEs

Consensus was reached for the majority of statements (31/49 statements) developed in relation to cutaneous AEs and included guidance on how to manage specific cutaneous AEs (Table 2).

**Table 2.** Expert consensus on incidence, severity and management of cutaneous AEs in pediatric patients treated with MAPKi therapies.

Consensus statement		Level of consensus (%)
*Incidence and severity of cutaneous AEs*		
17	Cutaneous AEs present the most common and significant management challenge with MAPKi therapies, especially MEK inhibitors	84
18	Nearly all patients on MAPKi therapy will experience at least one cutaneous reaction during the course of treatment	79
19	Cutaneous AEs are the most frequent factor impacting patient quality of life with MAPKi therapies	80
20	Cutaneous AEs have been reported with all types of MAPKi therapy, but certain cutaneous AEs can be strongly correlated with a specific type	76
21	Cutaneous AEs in pediatric patients can present within the first few weeks of treatment, and proactive management is essential to ensure treatment adherence	82
22	Tools that can be used to assess the severity of cutaneous reactions to MAPKi therapy include CTCAE, quality of life index, visual analogue scale, type of reaction, extent of rash, symptoms (itching, pain, etc), patient’s report, and limitations on activities of daily living.	85
23	The use of CTCAE alone may not accurately measure the severity of cutaneous reactions to MAPKi therapy, as it does not take into account the impact of the AE on the patient	83
24	Better methods are needed to measure the severity of cutaneous reactions to MAPKi therapy	79
25	In a clinical trial setting, a dermatologist or a clinician trained in identifying, grading, and managing cutaneous AEs should be involved in categorizing cutaneous AEs, in order to improve the consistency and accuracy of how these AEs are reported	79
26	A multidisciplinary approach (eg, pediatric oncologists and dermatologists with experience managing patients treated with MAPKi therapy if available), is encouraged for managing cutaneous AEs in pediatric patients treated with MAPKi therapy, especially when considering dose modifications or treatment interruptions	83
*Risk factors for cutaneous AEs*		
27	Pediatric patients with a history of atopic dermatitis/eczema may be at increased risk of exacerbated dermatologic reactions while on MAPKi therapies	76
28	Patients (particularly pubertal/post-pubertal patients) with a history of acneiform rash/acne may be at increased risk of exacerbated dermatologic reactions while on MAPKi therapies	77
29	Proactive management of pre-existing skin conditions, including topical treatments and close dermatologic monitoring, is essential in preventing severe AEs in high-risk patients	83
30	Adolescents may present with acneiform eruptions more frequently than pre-pubertal patients, which can be distressing and negatively impact adherence to therapy	87
*General skin care management*		
31	Proactive skin care is essential in preventing and managing skin AEs following exposure to MAPKi therapies, and may allow for avoidance of dose interruptions and reductions	82
32	Early initiation of prophylactic skin care measures is advised for all pediatric patients receiving MAPKi therapies, especially those with identified risk factors, such as age or a predisposing skin condition	77
33	Appropriate prophylactic skin care measures include gentle skin cleansers, emollients, and sun protection, including high-SPF sunscreen, avoiding excessive sun exposure, and/or to wear protective clothing (eg, hat, sunglasses, long sleeves, and trousers)	87
34	If available, it is preferable that a dermatologist with experience managing patients treated with MAPKi therapies is easily accessible to patients who experience cutaneous reactions to MAPKi therapy	82
35	The following may be prescribed to patients who are experiencing cutaneous reactions to MAPKi therapy: dry skin care (gentle soap, moisturizer), sun protection, dilute bleach baths, topical antibiotics, oral antibiotics, topical corticosteroids, topical retinoids, or oral retinoids	80
*Eczematous rash*		
36	Patients should moisturize twice daily with an emollient to prevent and manage an eczematous rash	80
37	If patients have a symptomatic eczematous rash, they should use a low-potency topical corticosteroid	79
38	If a symptomatic eczematous rash is not resolved by a low-potency topical corticosteroid, consider changing to a more potent topical corticosteroid	81
39	If a symptomatic eczematous rash is not controlled with a potent topical corticosteroid and dermatologist-guided approaches to manage rash are unsuccessful, MAPKi dose reduction should be considered	79
*Acneiform rash*		
40	For patients with severe acneiform rashes, clinicians should consult a dermatologist with experience with MAPKi, if available, and consider using semisynthetic oral tetracyclines	81
41	Early dermatologic intervention, and counseling if appropriate, may help reduce the psychosocial burden of acneiform rash, particularly in adolescents	83
*Paronychia*		
42	To prevent paronychia, patients should try to prevent potential trauma to the hands and feet (eg, avoiding tight shoes) and be advised on nail management (eg, not cutting the nails too short, avoiding cutting close to the nail fold)	83
43	For all severities of paronychia, the affected nail should be soaked regularly using an antiseptic	77
44	Infection should be managed with an antiseptic, with or without a topical antibiotic, according to the severity of paronychia and accompanying symptoms	78
45	If corticosteroid or antibiotic management is not successful, dose reduction or discontinuation of MAPKi should be considered if clinically appropriate	78
46	If multiple nails are affected in severe paronychia, it is recommended that patients seek consultation for expert podiatry or surgical management and consider dose reduction or discontinuation of MAPKi if clinically appropriate	80
47	To prevent further episodes of paronychia, the treating clinician should consider referral to a specialist (podiatrist, podologist, dermatologist, or pediatric surgeon, etc) if available	78

Abbreviations: AEs, adverse events; CTCAE, Common Terminology Criteria for Adverse Events; MAPKi, MAPK inhibitors; SPF, sun protection factor.

The literature showed, and experts agreed, that cutaneous-related AEs were the most commonly observed and the most challenging to manage. In a pediatric population, effective management of cutaneous AEs may be particularly important as the psychosocial impact could negatively affect treatment compliance, particularly in adolescents.

A number of tools are available to assess the severity and impact of cutaneous AEs; these include the Common Terminology Criteria for Adverse Events (CTCAE), quality of life indices, visual analogue scale, categorization of AE based on type or extent of reaction, recording of associated symptoms (itching, pain, etc), and limitations on activities of daily living. However, evaluating cutaneous AEs with several tools, as may be done in a clinical trial setting, may not be feasible in routine clinical practice. The accuracy of categorizing cutaneous AEs would be improved if performed by a dermatologist or trained HCP, which would be especially beneficial in clinical trials to ensure that the AE profile is well defined.

The CTCAE grading alone does not adequately assess the patient impact of the AE, which can be substantial, and, therefore, may not be considered an accurate representation of severity. As such, experts agreed that more effective methods to evaluate severity of cutaneous AEs with MAPKis are needed.

The value of including dermatology in the multidisciplinary team treating patients with pLGG was recognized and is recommended. In particular, whether the provider is specialized in pediatric dermatology or not, access to a dermatologist with experience managing patients treated with MAPKis is recommended, if available. The oncologist and dermatologist should work together to effectively manage cutaneous AEs and make decisions around dose modifications and treatment interruptions that may be required with MAPKi treatment.

#### Risk Factors

Experts agreed that a history of eczema or acne may make a patient more susceptible to experiencing exacerbated dermatologic conditions when treated with MAPKi therapies. In addition, there was consensus that pubertal and postpubertal children are more likely than prepubertal children to have acneiform eruptions. Proactive management and monitoring of at-risk patients may minimize the risk of severe cutaneous AEs.

#### General Skin Care Management

The experts recognized that the different MAPKi treatments have differing cutaneous AE profiles; however, the overlap was considered sufficient to warrant developing general guidance on skin care and recommendations for treating eczematous rash, acneiform rash, and paronychia. Patients who experience cutaneous AEs in response to MAPKi therapy should be prescribed treatments for dry skin (gentle cleansers and emollients), sun protection, dilute bleach baths, topical or oral antibiotics, topical corticosteroids, and topical or oral retinoids, dependent on the nature of the AE.

Early prophylactic skin care is recommended for all pediatric patients treated with MAPKi therapies, not just those considered at risk due to their age or a preexisting dermatologic condition. A proactive approach to skin care, such as moisturization and sun protection, is essential for managing cutaneous AEs and to minimize the need for dose changes or treatment interruptions.

#### Eczematous Rash

To prevent and manage eczematous rash, moisturizing twice daily with an emollient is recommended. If the rash is symptomatic (dry, itchy, inflamed), a low-potency topical corticosteroid should be used. If this does not resolve the symptoms, a more potent topical corticosteroid should be considered. The MAPKi dose reduction should only be considered when symptomatic eczematous rash cannot be controlled with a potent topical corticosteroid or other dermatologist-guided management strategies.

#### Acneiform Rash

If the acneiform rash is severe, clinicians should consult a dermatologist and consider prescribing semisynthetic oral tetracyclines if patient age allows. Early intervention to treat acneiform rash, including counseling if warranted, may help to reduce the associated psychosocial burden, particularly in adolescents who may be the most affected.

#### Paronychia

Paronychia (nail infection) is a relatively common AE with MAPKi treatment; therefore, prevention by advising patients on appropriate nail management and to avoid trauma to the hands and feet is recommended. If paronychia occurs, the infected nail should be soaked regularly with an antiseptic. A topical antibiotic may also be required depending on how severe the paronychia is and the accompanying symptoms. In severe cases where multiple nails are affected, consultation with podiatry or surgical management is recommended; dose reduction or discontinuation of MAPKi treatment may also be warranted, at the discretion of the treating clinician and if deemed clinically appropriate. In patients with a history of paronychia, referral to a specialist, for example, podiatry or dermatology, should also be considered.

### Noncutaneous AEs

The steering committee developed statements for noncutaneous AEs, including gastrointestinal, cardiac, ophthalmologic, and endocrine-related AEs, as well as laboratory abnormalities. Consensus was not achieved by the expert contributors for most statements (Table S9). Those statements that did achieve consensus are detailed in Table 3.

**Table 3.** Expert consensus on incidence, severity and management of noncutaneous AEs in pediatric patients treated with MAPKi therapies.

Consensus statement		Level of consensus (%)
*Endocrine-related AEs*		
48	Early signs of hyponatremia, such as headache, lethargy, nausea, and confusion, should prompt immediate evaluation, and serum electrolytes should be checked promptly	76
*Laboratory abnormalities*		
49	CPK levels may be elevated among patients taking MAPKi; in rare cases of symptomatic CPK elevation (primarily muscle pain and weakness) dose interruption or reduction should be considered	81
50	If CPK elevation is less than 5 times the upper limit of normal (ie, mild to moderate) and patients are asymptomatic, MAPKi should not be discontinued	79

Abbreviations: AEs, adverse events; CPK, creatinine phosphokinase; MAPKi, MAPK inhibitors.

Symptoms such as headache, lethargy, nausea, and confusion that could be early signs of hyponatremia should prompt immediate evaluation; serum electrolytes should also be measured promptly. Mild-to-moderate elevation in CPK (<5 × upper limit of normal) is not of concern if no symptoms are present. In the rare event of symptomatic CPK elevation, which is characterized mainly by muscle pain and weakness, interrupting MAPKi treatment or reducing the dose should be considered.

## Discussion

A modified Delphi approach was used to develop a consensus on the management of AEs in pediatric patients with pLGG treated with MAPKi therapies. The aim was to identify common AEs that occur with MAPKi therapies and to provide guidance on their prevention and management that can be adopted in patients with pLGG, to minimize dose modifications or interruptions. Data on AEs with MAPKi therapies in pediatric cancers were identified by a comprehensive literature search, and this was used by a steering committee to develop statements. A large, global, multidisciplinary group of experts, including some from low- and middle-income countries, participated in surveys to determine consensus recommendations with a high participation rate.

The nature and severity of AEs reported with MAPKi therapies in pediatric cancers were quite broad; however, AEs were rarely life-threatening with few fatal AEs reported in clinical studies.^4^^,^^6^^,^^8^^,^^12^^,^^16–26^ Specifically in patients with pLGG, 2 fatal AEs were reported in a phase 2 study of tovorafenib, both were due to disease progression and not considered related to study drug.^6^ In trials of dabrafenib and trametinib, alone or in combination,^4^^,^^16^^,^^19^ or selumetinib, there were no treatment-related deaths reported in patients with pLGG.^12^^,^^20^^,^^24^

It is important to note that there are key drug class differences in terms of AE profiles, and clinicians treating patients with pLGG need to be aware of these differences and should follow local guidance. While most toxicities of MAPKi therapies are thought to be a direct effect of MAPKi inhibition, type I BRAFi are selective mutant inhibitors, which can proceed to paradoxical activation of the MAPK pathway resulting in secondary skin malignancy;^27^^,^^28^ this is not seen with type II RAFi and MEKi. Combining a type I BRAFi and an MEKi has been shown to improve tolerability, as reported in patients with pLGG with a BRAF V600E mutation.^19^ Similarly, reductions in growth velocity have only been observed in patients with pLGG treated with tovorafenib.^6^^,^^29^ Data available to date indicate that this growth suppression is reversed posttreatment, with catch-up growth reported in most cases;^29^ however, long-term data are needed to understand the true impact.

Nearly all patients on MAPKi therapies experience cutaneous AEs,^19^^,^^22^^,^^23^ with the incidence up to 96% of patients being affected in clinical studies.^23^ Cutaneous AEs reported with these treatments include photosensitivity, pruritus, xerosis, eczematous rash, acneiform rash, maculopapular rash, skin papilloma, hyperkeratosis, paronychia, hair thinning, and hair color changes.^7^^,^^30–35^ Cutaneous AEs can present early in MAPKi treatment,^36–46^ can have a significantly negative impact on patients’ quality of life,^36^ and can be difficult to manage.

Different cutaneous AEs have been observed with different classes of MAPKi therapies. In a retrospective analysis of pediatric patients treated with type 1 BRAFi and/or MEKi, keratosis pilaris–like reaction, photosensitivity, palmoplantar hyperkeratosis, eruptive nevi, and panniculitis were most likely to occur with BRAFi treatment or combination therapy compared with MEKi treatment alone. In contrast, MEKi treatment was more often associated with acneiform eruption, dermatitis, and paronychia.^47^ Hair color changes are the most common AE occurring with the type I RAFi, tovorafenib, affecting 76% of patients in the phase 2 trial;^6^ this AE has also been reported in some studies with MEKi, reportedly affecting 9%-68% of pediatric patients.^18^^,^^36^^,^^37^^,^^48^ The combination of trametinib (MEKi) and dabrafenib (BRAFi) resulted in fewer incidences of paronychia and papular rash than trametinib alone in patients with pLGG with a BRAF V600E mutation in a phase 1/2 trial. Notably, in the trial, the proportion of patients discontinuing treatment due to an AE was lower in the combination therapy treatment group (22.2%) than the trametinib monotherapy group (53.8%).^19^ Consensus was reached on general skin care and management of specific cutaneous AEs across MAPKi therapies in patients with pLGG despite these drug class differences.

There is substantial evidence that dose interruptions may be required to manage AEs from clinical studies of MAPKi therapies.^6^^,^^8^^,^^11^^,^^20^^,^^22^^,^^25^ Cutaneous AEs may not respond to initial management strategies or require multiple interventions, and cutaneous AEs have also resulted in MAPKi treatment having to be discontinued.^38^^,^^42^^,^^43^^,^^45^^,^^49–51^

Other common AEs reported with MAPKi therapies are diarrhea, nausea, vomiting, abdominal pain, constipation, stomatitis, headache, fatigue, dyspnea, cough, pyrexia, infections, edema, hemorrhage, musculoskeletal pain, arthralgia, visual impairment, left ventricular dysfunction, and chills.^7^^,^^30–35^^,^^52^ In this consensus initiative, recommendations for gastrointestinal, cardiac, endocrine, and ophthalmologic AEs among others were also considered; however, consensus on the related statements was limited. In general, these AEs were drug class–specific or drug-specific, were only seen rarely, or more data are needed to fully understand their impact and how they can be managed.

Chronic pLGG can be challenging to manage, particularly when the tumor is located in the suprasellar region, which limits surgery as a treatment option. In such cases, the tumor and/or its surgical management can lead to hypothalamic-pituitary dysfunction with obesity and hormonal imbalances, including arginine vasopressin (AVP) deficiency (also known as central diabetes insipidus). Hypothalamic dysfunction can result in memory impairment, attention deficit, and reduced impulse control, and also increase the risk of cardiovascular and metabolic disorders in these patients.^53^ Systemic treatment options are therefore needed in patients with suprasellar tumors.

In patients with pLGG and AVP deficiency, hyponatremia has been reported during treatment with BRAFi/MEKi.^54^^,^^55^ As such, when administering MAPKi to a patient with known hypothalamic-pituitary dysfunction, the pediatric endocrinologist should be consulted for advice on sodium monitoring and to provide tools for management of fluid balances in the home setting.^55^ It is also important that for all patients with pLGG in the suprasellar region, treatment should be started in consultation with the pediatric endocrinologist for adequate monitoring and guidance of growth and serum sodium values. Future studies are needed to understand the effect of MAPKis on linear growth and body mass index.

In a recent review from Crotty et al,^15^ the authors discussed MAPKi-related toxicity in pediatric patients and provided some guidance on treatment and monitoring based on data from clinical studies. In this Delphi consensus, guidance on managing rash and paronychia is based on not only the available literature but also the experience and expertise of more than 90 HCPs. That being said, the consensus statements for rash and paronychia are broadly aligned with Crotty et al. For elevated CPK, Crotty et al recommend dose interruption and then resuming treatment at a reduced dose, with regular CPK monitoring. Elevated CPK levels have been reported in pediatric patients taking MAPKis, although dose interruption or reduction is not typically needed.^6^^,^^8^^,^^12^^,^^17^^,^^18^^,^^20^^,^^24^^,^^25^ As such, the recommendation from the consensus is to only consider these changes to treatment if symptoms are present with the elevated CPK.

Decreased cardiac function, as noted with BRAFi and MEKi in clinical studies, and retinal toxicity with selumetinib were also highlighted by Crotty et al.^15^ Consensus was not achieved as to how to manage these AEs among the group of global experts convened. This may be because a high proportion of the contributors were oncologists, with relatively few experts from other specialties participating in the surveys. Oncologists may rely on other specialties to advise them as to how to manage AEs that occur infrequently or are outside of their area of expertise. An awareness of these potential, albeit often rare, AEs is important when starting patients on an MAPKi treatment to identify toxicity, and to minimize the need to reduce doses or discontinue treatment altogether. The importance of having a multidisciplinary team taking care of patients with pLGG being treated with MAPKi therapies was also highlighted in this consensus initiative. In addition, patient and caregiver education on AEs was also highlighted here as important for managing their expectations of MAPKi treatment, with the goal of achieving adherence.

The potential long-term health impacts of MAPKi treatment remain unknown, so long-term monitoring of these patients is needed.^15^ The use of MAPK-directed therapies in patients with pLGG is increasing, in both the clinical trial setting and routine practice. The combination therapy of trametinib and dabrafenib is already approved as a first-line treatment for pLGG,^31^ and tovorafenib and selumetinib are currently being investigated as first-line treatments.^56–58^ Duration of exposure to these treatments can also be lengthy, with patients typically receiving treatment for more than a year.^4^^,^^6^ This initiative identified gaps in understanding of the incidence and management of the broad spectrum of AEs that can occur with MAPKi therapies in children. Understanding the AEs that can arise and how they can be effectively managed will be an ongoing endeavor, with more research needed so that the evidence-based recommendations presented here can be expanded upon. The toxicity of the MEKi, selumetinib, which is indicated in pediatric patients with NF1 who have symptomatic, inoperable plexiform neurofibromas,^34^ was recently evaluated in the Food and Drug Administration Adverse Event Reporting System (FAERS) database.^59^ The AEs observed were consistent with safety findings in the selumetinib clinical studies, with cardiac, ophthalmologic, and cutaneous AEs being reported in routine clinical practice.^59^ Ongoing surveillance for MAPKi therapies is warranted to fully understand the toxicity profile of these treatments in pLGG. While it is currently not possible to predict which patients will develop significant toxicities in response to MAPKi therapies, pharmacogenomics studies could be conducted to identify predictive markers for AEs, as demonstrated with chemotherapies.^60^ Using such data could potentially minimize the need for treatment discontinuation or dose reductions when using these promising targeted therapies in the treatment of pLGG.

Limitations of this initiative include most contributors being oncologists with a limited number of experts from other specialties, and experts being predominantly from the United States and Europe. Whether the recommendations provided here are feasible at a global level is unknown; centers in some countries may have fewer resources or lack access to multiple specialties, but use of MAPKi is emerging in low-to-middle-income countries.^61^ Also, the AEs considered were based on the available literature, with a focus on common AEs; therefore, this was not an exhaustive review of all AEs reported with MAPKi therapies. Limited data exist on some AEs occurring with MAPKi treatment; therefore, there was not enough evidence or experience in treating these AEs among the contributors to achieve consensus on evidence-based recommendations.

## Conclusion

The development of these consensus statements provides best practice guidance on the management of common AEs in patients with pLGG treated with MAPKi therapies. Sharing these recommendations among HCPs will hopefully lead to patients with pLGG achieving optimal benefit from MAPKi therapies while minimizing and effectively managing AEs.

## Supplementary Material

npag006_Supplementary_Data

## Data Availability

All data are contained within the manuscript.
