# Supplementary material for: Management of adverse events in patients with pediatric low-grade glioma treated with MAPK-directed therapies: Delphi consensus recommendations and unmet needs
Source: Neurooncol Pract. 2026 Feb 6;13(3):620–33. doi: 10.1093/nop/npag006 (PMC13161904; doi:10.1093/nop/npag006)
Supplement: npag006_Supplementary_Data [file npag006_supplementary_data.docx]

# Management of adverse events in patients with pediatric low-grade glioma treated with MAPK-directed therapies: Delphi consensus recommendations and unmet needs

Darren Hargrave^1,2^, Daniel C. Bowers^3^, Stewart Goldman^4^, Grant T. Liu^5^, Jennifer T. Huang^6^,
Hanneke M. van Santen^7^, Nathan J. Robison^8^, Michal Zapotocky^9,10^, Eric Bouffet^11^.

^1^Great Ormond Street Hospital for Children, London, UK; ^2^UCL Great Ormond Street Institute of Child Health, London, UK; ^3^Simmons Comprehensive Cancer Center and the Department of Pediatrics, University of Texas Southwestern Medical Center, Dallas, TX, USA; ^4^Department of Child Health, University of Arizona College of Medicine- Phoenix, Phoenix Children’s Hospital, Phoenix, AZ, USA; ^5^Division of Ophthalmology, Children’s Hospital of Philadelphia, Philadelphia, PA, USA; ^6^Dermatology Section, Boston Children’s Hospital, Boston, MA, USA; ^7^Princess Máxima Center for Pediatric Oncology and Department of Pediatric Endocrinology, Wilhelmina Children's Hospital, UMC, Utrecht, Netherlands; ^8^Cancer and Blood Disease Institute, Children's Hospital Los Angeles, Los Angeles, CA, USA; ^9^Department of Paediatric Haematology and Oncology, Second Faculty of Medicine, Charles University, Prague, Czech Republic; ^10^Center for Pediatric Neuro-oncology, Motol University Hospital, Prague, Czech Republic; ^11^Division of Hematology/Oncology, University of Toronto, Hospital for Sick Children (SickKids), Toronto, ON, Canada

# Supplementary Material

## Supplementary Methods

### Literature Search and Evidence Grading

- PubMed, Web of Science and Cochrane database search terms relating to MAPKi, safety and tolerability, and pediatric patient populations are provided in Supplementary Tables 1–4
- The following congresses were searched using the key terms shown in Table S5: ASCO: 2021–2023, ASPHO: 2021–2023, ESMO: 2021–2023, Global NF conference:
  2019–2023, ISPNO: 2018–2022, SIOP: 2021–2023, SNO: 2021–2023, Pediatric SNO: 2019–2023
- Literature search results were screened based on the relevance of the title and abstract following the inclusion and exclusion criteria provided in Supplementary Table 6
- All articles/abstracts considered relevant for data extraction were graded according to their level of evidence using a modified version of the Jovell/Navarro-Rubio criteria) [Supplementary Table 7]^1^
- Publications used to aid in drafting statements are captured in Supplementary Table 8

### References

**1.** Jovell AJ, Navarro-Rubio MD. [Evaluation of scientific evidence]. *Med Clin (Barc).* 1995; 105(19):740–743.

### **Supplementary Table 1.** PubMed Search Strategy

| **Search Number** | **Query** | **Filters** | **Results** | **Notes** |
| --- | --- | --- | --- | --- |
| 1 | "MAPK inhibitor*"[all] OR "map kinase inhibitor*"[all] OR "RAF inhibitor*"[all] OR "BRAF inhibitor*"[all] OR "CRAF inhibitor*"[all] OR "MEK inhibitor*"[all] OR "MEK1/2 inhibitor*"[all] |  | 15,652 | MAPK inhibitors |
| 2 | "Vemurafenib"[Mesh] OR vemurafenib[all] OR PLX4032[all] OR "PLX-4032"[all] OR "PLX 4032"[all] OR RG7204[all] OR "RG-7204"[all] OR "RG 7204"[all] OR Zelboraf[all] OR R05185426[all] |  | 3,039 |  |
| 3 | "dabrafenib" [Supplementary Concept] OR dabrafenib[all] OR "GSK 2118436"[all] OR "GSK-2118436"[all] OR GSK2118436[all] |  | 1,902 |  |
| 4 | "encorafenib" [Supplementary Concept] OR encorafenib[all] OR LGX818[all] OR "LGX-818"[all] OR "LGX 818"[all] |  | 364 |  |
| 5 | "tovorafenib" [Supplementary Concept] OR tovorafenib[all] OR "TAK-580"[all] OR "TAK580"[all] OR TAK580[all] OR "MLN 2480"[all] OR "MLN-2480"[all] OR MLN2480[all] |  | 14 |  |
| 6 | "trametinib" [Supplementary Concept] OR trametinib[all] OR "JTP 74057"[all] OR "JTP-74057"[all] OR JTP74057[all] OR "GSK 1120212"[all] OR "GSK-1120212"[all] OR GSK1120212[all] |  | 2,358 |  |
| 7 | "N-(2,3-dihydroxypropyl)-1-((2-fluoro-4-iodophenyl)amino)isonicotinamide" [Supplementary Concept] OR pimasertib[all] OR "MSC-1236369B"[all] OR "MSC 1236369B"[all] OR MSC1236369B[all] OR "AS 703026"[all] OR "AS-703026"[all] OR AS703026[all] |  | 66 |  |
| 8 | "binimetinib" [Supplementary Concept] OR binimetinib[all] OR Mektovi[all] OR MEK162[all] OR "MEK 162"[all] OR "MEK-162"[all] |  | 414 |  |
| 9 | "cobimetinib" [Supplementary Concept] OR cobimetinib[all] OR Cotellic[all] OR "GDC-0973"[all] OR "GDC 0973"[all] OR GDC0973[all] OR XL518[all] OR "XL 518"[all] OR "XL-518"[all] |  | 500 |  |
| 10 | "AZD 6244" [Supplementary Concept] OR "AZD 6244"[all] OR "AZD-6244"[all] OR AZD6244[all] OR selumetinib OR "ARRY 142886"[all] OR "ARRY-142886 "[all] OR ARRY142886[all] |  | 905 |  |
| 11 | "mirdametinib" [Supplementary Concept] OR mirdametinib[all] OR "PD 0325901"[all] OR "PD-0325901"[all] OR PD0325901[all] OR "PD 325901"[all] OR "PD-325901"[all] OR PD325901[all] |  | 553 |  |
| 12 | #1 OR #2 OR #3 OR #4 OR #5 OR #6 OR #7 OR #8 OR #9 OR #10 OR #11 |  | 19,204 |  |
| 13 | "Drug-Related Side Effects and Adverse Reactions"[Mesh] OR "adverse reaction*"[all] OR "adverse drug reaction*"[all] OR "adverse event*"[all] OR "adverse drug event*"[all] OR "adverse effect*"[all] OR "adverse drug effect*"[all] OR "side effect*"[all] OR toxicit*[all] |  | 3,307,022 | Safety and tolerability |
| 14 | #12 AND #13 |  | 3,398 |  |
| 15 | "Pediatrics"[Mesh] OR Pediatric*[all] OR Paediatric*[all] OR "Child"[Mesh] OR child[all] OR children[all] OR "Young Adult"[Mesh] OR "young adult*"[all] OR "Adolescent"[Mesh] OR Adolescent*[all] OR adolescence[all] OR teen[all] OR teens[all] OR teenager*[all] OR youth[all] OR youths[all] |  | 5,289,514 | Pediatric patient populations |
| 16 | #14 AND #15 |  | 409 |  |
| 17 | #14 AND #15 | from 2014/1/1 - 3000/12/12 | 403 |  |

### **Supplementary Table 2.** Web of Science Core Collection Search Strategy

| **Web of Science Core Collection** | | | |
| --- | --- | --- | --- |
| **Search Number** | **Query** | **Results** | **Notes** |
| 1 | ALL=("MAPK inhibitor*" OR "map kinase inhibitor*" OR "RAF inhibitor*" OR "BRAF inhibitor*" OR "CRAF inhibitor*" OR "MEK inhibitor*" OR "MEK1/2 inhibitor*") | 17289 | MAPK inhibitors |
| 2 | ALL=(vemurafenib OR PLX4032 OR "PLX-4032" OR "PLX 4032" OR RG7204 OR "RG-7204" OR "RG 7204" OR Zelboraf OR R05185426) | 4913 |  |
| 3 | ALL=(dabrafenib OR "GSK 2118436" OR "GSK-2118436" OR GSK2118436) | 3403 |  |
| 4 | ALL=(encorafenib OR LGX818 OR "LGX-818" OR "LGX 818") | 519 |  |
| 5 | ALL=(tovorafenib OR "TAK-580" OR "TAK580" OR TAK580 OR "MLN 2480" OR "MLN-2480" OR MLN2480) | 36 |  |
| 6 | ALL=(trametinib OR "JTP 74057" OR "JTP-74057" OR JTP74057 OR "GSK 1120212" OR "GSK-1120212" OR GSK1120212) | 3547 |  |
| 7 | ALL=("N-(2,3-dihydroxypropyl)-1-((2-fluoro-4-iodophenyl)amino)isonicotinamide" OR pimasertib OR "MSC-1236369B" OR "MSC 1236369B" OR MSC1236369B OR "AS 703026" OR "AS-703026" OR AS703026) | 96 |  |
| 8 | ALL=(binimetinib OR Mektovi OR MEK162 OR "MEK 162" OR "MEK-162") | 596 |  |
| 9 | ALL=(cobimetinib OR Cotellic OR "GDC-0973" OR "GDC 0973" OR GDC0973 OR XL518 OR "XL 518" OR "XL-518") | 710 |  |
| 10 | ALL=("AZD 6244" OR "AZD-6244" OR AZD6244 OR selumetinib OR "ARRY 142886" OR "ARRY-142886 " OR ARRY142886) | 1381 |  |
| 11 | ALL=(mirdametinib OR "PD 0325901" OR "PD-0325901" OR PD0325901 OR "PD 325901" OR "PD-325901" OR PD325901) | 517 |  |
| 12 | #1 OR #2 OR #3 OR #4 OR #5 OR #6 OR #7 OR #8 OR #9 OR #10 OR #11 | 23465 |  |
| 13 | ALL=("adverse reaction*" OR "adverse drug reaction*" OR "adverse event*" OR "adverse drug event*" OR "adverse effect*" OR "adverse drug effect*" OR "side effect*" OR toxicit*) | 1244205 | Safety and tolerability |
| 14 | #12 AND #13 | 2740 | MAPK inhibitors and safety and tolerability |
| 15 | ALL=(Pediatric* OR Paediatric* OR child OR children OR "young adult*" OR "Adolescent" OR Adolescent* OR adolescence OR teen OR teens OR teenager* OR youth OR youths) | 2826989 | Pediatric patient populations |
| 16 | #14 AND #15 | 181 | All terms |
| 17 | #14 AND #15 Timespan: 2014-01-01 to 2024-04-22 | 180 | Date filter |
| 18 | Deduplication step using PMIDs from PubMed search | 387 | Deduplication |
| 19 | #17 NOT #18 | 43 🡪 42 |  |

### **Supplementary Table 3.** Web of Science BIOSIS Search Strategy

| **Web of Science BIOSIS** | | | |
| --- | --- | --- | --- |
| **Search Number** | **Query** | **Results** | **Notes** |
| 1 | TS=("MAPK inhibitor*" OR "map kinase inhibitor*" OR "RAF inhibitor*" OR "BRAF inhibitor*" OR "CRAF inhibitor*" OR "MEK inhibitor*" OR "MEK1/2 inhibitor*") | 15563 | MAPK inhibitors |
| 2 | TS=(vemurafenib OR PLX4032 OR "PLX-4032" OR "PLX 4032" OR RG7204 OR "RG-7204" OR "RG 7204" OR Zelboraf OR R05185426) | 2392 |  |
| 3 | TS=(dabrafenib OR "GSK 2118436" OR "GSK-2118436" OR GSK2118436) | 1148 |  |
| 4 | TS=(encorafenib OR LGX818 OR "LGX-818" OR "LGX 818") | 199 |  |
| 5 | TS=(tovorafenib OR "TAK-580" OR "TAK580" OR TAK580 OR "MLN 2480" OR "MLN-2480" OR MLN2480) | 11 |  |
| 6 | TS=(trametinib OR "JTP 74057" OR "JTP-74057" OR JTP74057 OR "GSK 1120212" OR "GSK-1120212" OR GSK1120212) | 1829 |  |
| 7 | TS=("N-(2,3-dihydroxypropyl)-1-((2-fluoro-4-iodophenyl)amino)isonicotinamide" OR pimasertib OR "MSC-1236369B" OR "MSC 1236369B" OR MSC1236369B OR "AS 703026" OR "AS-703026" OR AS703026) | 85 |  |
| 8 | TS=(binimetinib OR Mektovi OR MEK162 OR "MEK 162" OR "MEK-162") | 288 |  |
| 9 | TS=(cobimetinib OR Cotellic OR "GDC-0973" OR "GDC 0973" OR GDC0973 OR XL518 OR "XL 518" OR "XL-518") | 374 |  |
| 10 | TS=("AZD 6244" OR "AZD-6244" OR AZD6244 OR selumetinib OR "ARRY 142886" OR "ARRY-142886 " OR ARRY142886) | 970 |  |
| 11 | TS=(mirdametinib OR "PD 0325901" OR "PD-0325901" OR PD0325901 OR "PD 325901" OR "PD-325901" OR PD325901) | 605 |  |
| 12 | #1 OR #2 OR #3 OR #4 OR #5 OR #6 OR #7 OR #8 OR #9 OR #10 OR #11 | 18779 |  |
| 13 | TS=("adverse reaction*" OR "adverse drug reaction*" OR "adverse event*" OR "adverse drug event*" OR "adverse effect*" OR "adverse drug effect*" OR "side effect*" OR toxicit*) | 980797 | Safety and tolerability |
| 14 | #12 AND #13 | 1683 | MAPK inhibitors and safety and tolerability |
| 15 | TS=(Pediatric* OR Paediatric* OR child OR children OR "young adult*" OR "Adolescent" OR Adolescent* OR adolescence OR teen OR teens OR teenager* OR youth OR youths) | 1313587 | Pediatric patient populations |
| 16 | #14 AND #15 | 121 | All terms |
| 17 | #14 AND #15 Timespan: 2014-01-01 to 2024-04-22 | 118 | Date filter |
| 18 | Deduplication step using PMIDs from PubMed search | 189 | Deduplication |
| 19 | #17 NOT #18 | 53 🡪 51 |  |

### **Supplementary Table 4.** Cochrane Database Search Strings

| **Search number** | **Query** | **Results** | **Notes** |
| --- | --- | --- | --- |
| 1 | (MAPK NEXT inhibitor*) OR ("map kinase" NEXT inhibitor*) OR (RAF NEXT inhibitor*) OR (BRAF NEXT inhibitor*) OR (CRAF NEXT inhibitor*) OR (MEK NEXT inhibitor*) OR (MEK1/2 NEXT inhibitor*) | 648 | MAPK inhibitors |
| 2 | [mh Vemurafenib] | 81 |  |
| 3 | vemurafenib OR PLX4032 OR "PLX-4032" OR "PLX 4032" OR RG7204 OR "RG-7204" OR "RG 7204" OR Zelboraf OR R05185426 | 274 |  |
| 4 | dabrafenib OR "GSK 2118436" OR "GSK-2118436" OR GSK2118436 | 311 |  |
| 5 | encorafenib OR LGX818 OR "LGX-818" OR "LGX 818" | 162 |  |
| 6 | tovorafenib OR "TAK-580" OR "TAK580" OR TAK580 OR "MLN 2480" OR "MLN-2480" OR MLN2480 | 4 |  |
| 7 | trametinib OR "JTP 74057" OR "JTP-74057" OR JTP74057 OR "GSK 1120212" OR "GSK-1120212" OR GSK1120212 | 382 |  |
| 8 | N-(2,3-dihydroxypropyl)-1-((2-fluoro-4-iodophenyl)amino)isonicotinamide OR pimasertib OR "MSC-1236369B" OR "MSC 1236369B" OR MSC1236369B OR "AS 703026" OR "AS-703026" OR AS703026 | 22 |  |
| 9 | binimetinib OR Mektovi OR MEK162 OR "MEK 162" OR "MEK-162" | 182 |  |
| 10 | cobimetinib OR Cotellic OR "GDC-0973" OR "GDC 0973" OR GDC0973 OR XL518 OR "XL 518" OR "XL-518" | 168 |  |
| 11 | AZD 6244 OR "AZD-6244" OR AZD6244 OR selumetinib OR "ARRY 142886" OR "ARRY-142886 " OR ARRY142886 | 176 |  |
| 12 | mirdametinib OR "PD 0325901" OR "PD-0325901" OR PD0325901 OR "PD 325901" OR "PD-325901" OR PD325901 | 7 |  |
| 13 | #1 OR #2 OR #3 OR #4 OR #5 OR #6 OR #7 OR #8 OR #9 OR #10 OR #11 OR #12 | 1264 |  |
| 14 | [mh "Drug-Related Side Effects and Adverse Reactions"] | 5197 | Safety and tolerability |
| 15 | (adverse NEXT reaction*) OR ("adverse drug" NEXT reaction*) OR (adverse NEXT event*) OR ("adverse drug" NEXT event*) OR (adverse NEXT effect*) OR ("adverse drug" NEXT effect*) OR (side NEXT effect*) OR toxicit* | 463077 |  |
| 16 | #14 OR #15 | 463472 | MAPK inhibitors and safety and tolerability |
| 17 | #13 AND #16 | 697 |  |
| 18 | [mh "Pediatrics"] OR [mh "Child"] OR [mh "Young Adult"] OR [mh "Adolescent"] | 227825 | Pediatric populations |
| 19 | Pediatric* OR Paediatric* OR child OR children OR (young NEXT adult*) OR Adolescent* OR adolescence OR teen OR teens OR teenager* OR youth OR youths | 403944 |  |
| 20 | #18 OR #19 | 403989 | All terms |
| 21 | #17 AND #20 | 118 |  |
| 22 | #17 AND #20 - 2014-2024 | 117 | Date filter |
| N/A | Deduplication | 51 | Deduplication |

### **Supplementary Table 5.** Keywords for Congress Abstract Search

| **Search Topic** | **Key Terms** |
| --- | --- |
| **MAPKi Terms** | **General:** MAPK inhibitor; RAF inhibitor; BRAF inhibitor; CRAF inhibitor; MEK inhibitor; MEK1/2 inhibitor; MAPK inhibitors; RAF inhibitors; BRAF inhibitors; CRAF inhibitors; MEK inhibitors; MEK1/2 inhibitors  **Drug-specific:** Vemurafenib; PLX4032; PLX-4032; PLX 4032; RG7204; RG-7204; RG 7204; Dabrafenib; GSK 2118436; GSK-2118436; GSK2118436; Encorafenib; LGX818; LGX-818; LGX 818; Tovorafenib; TAK‑580; TAK580; TAK580; MLN 2480; MLN-2480; MLN2480; Trametinib; JTP 74057; JTP-74057; JTP74057; GSK 120212; GSK-1120212; GSK1120212; N-(2,3-dihydroxypropyl)-1-((2-fluoro-4 odophenyl)amino)isonicotinamide; Pimasertib; MSC-1236369B; MSC 1236369B; MSC1236369B; AS 03026; AS-703026; AS703026; Binimetinib; Mektovi; MEK162; MEK 162; MEK-162; Cobimetinib; Cotellic; GDC-0973; GDC 0973; GDC0973; XL518; XL 518; XL-518; AZD 6244; AZD-6244; AZD6244; Selumetinib; ARRY 142886; ARRY-142886; ARRY142886; Mirdametinib; PD 0325901; PD-0325901; PD0325901; PD 325901; PD-325901; PD325901 |
| **Safety and Tolerability Terms** | Drug-related side effects and adverse reactions; Adverse reaction; Adverse reactions; Adverse drug reaction; Adverse drug reactions; Adverse event; Adverse events; Adverse drug event; Adverse drug events; Adverse effect; Adverse effects; Adverse drug effect; Adverse drug effects; Side effect; Side effects; Toxicity; Toxicities |
| **Pediatric Patient Population Terms** | Pediatrics; Pediatrics; Paediatric; Paediatrics; Child; Children; Young adult; Young adults; Adolescent; Adolescents; Adolescence; Teen; Teens; Teenager; Teenagers; Youth; Youths |

### **Supplementary Table 6.** Inclusion and Exclusion Criteria

|  | Inclusion Criteria | Exclusion Criteria |
| --- | --- | --- |
| Study/Publication Type | - Prospective clinical trials (Phase 0, 1, Phase 1/2, Phase 2, Phase 3, Phase 4) - Retrospective clinical trials - Systematic reviews - Meta-analyses - Reviews - Guidelines/consensus statements - Congress materials - Case reports - English language - Publication date: 2014–2024 | - Preclinical studies - Study protocols - Studies >10 years old |
| Subjects/Clinical Setting | - Humans - Pediatric/adolescent/young adult patients (<25 years old) - Undergoing MAPK targeted therapy treatment | - Adult patients - Animal studies |

### **Supplementary Table 7.** Evidence Grading Criteria^1^

| **Level of Evidence** | **Type of Study** | **Strength of Evidence** |
| --- | --- | --- |
| Level 1 | Meta-analyses of randomized controlled trials | Good |
| Level 2 | Large-sample randomized controlled trials |  |
| Level 3 | Small-sample randomized controlled trials | Good to fair |
| Level 4 | Non-randomized controlled prospective trials |  |
| Level 5 | Non-randomized controlled retrospective trials |  |
| Level 6 | Cohort studies, non-randomized, uncontrolled prospective trials, or meta-analyses of studies of mixed design | Fair |
| Level 7 | Case-control studies |  |
| Level 8 | Non-controlled clinical series, descriptive studies or meta-analyses of case reports/series | Poor |
| Level 9 | Anecdotes or case reports |  |

### **Supplementary Table 8.** Relevant Articles and Congress Abstracts Identified

| **Authors** | **Title** | **Year** | **Journal** | **Level of Evidence** |  |
| --- | --- | --- | --- | --- | --- |
| **Journal Articles: Clinical Studies AND Case Reports/Series** | | | | | |
| Borgia P, Piccolo G, Santangelo A, *et al.* | Dermatologic effects of selumetinib in pediatric patients with neurofibromatosis type 1: clinical challenges and therapeutic management | 2024 | J Clin Med  DOI: 10.3390/jcm13061792 | 8 |  |
| Cournoyer E, Ferrell J, Sharp S, *et al.* | Dabrafenib and trametinib in Langerhans cell histiocytosis and other histiocytic disorders | 2024 | Haematologica  DOI: 10.3324/haematol.2023.283295 | 8 |  |
| Friedland R, Glucka M, Amitay-Laish I, *et al.* | Cutaneous reactions in pediatric patients treated with MEK inhibitors: a retrospective single-center study | 2024 | Dermatology  DOI: 10.1159/000539374 | 8 |  |
| Kilburn LB, Khuong-Quang DA, Hansford JR, *et al.* | The type II RAF inhibitor tovorafenib in relapsed/refractory pediatric low-grade glioma: the phase 2 FIREFLY-1 trial | 2024 | Nat Med  DOI: 10.1038/s41591-023-02668-y | 6 |  |
| Peacock BC, Tripathy S, Hanania HL, *et al.* | Cutaneous toxicities of mitogen-activated protein kinase inhibitors in children and young adults with neurofibromatosis-1 | 2024 | J Neurooncol  DOI: 10.1007/s11060-024-04617-2 | 8 |  |
| Ahmed F, Fisher MJ, Snyder KM, *et al.* | Adverse cutaneous reactions associated with MEK inhibitor therapy in a pediatric population | 2023 | J Am Acad Dermatol  DOI: 10.1016/j.jaad.2023.06.052 | 8 |  |
| Bouffet E, Geoerger B, Moertel C, *et al.* | Efficacy and safety of trametinib monotherapy or in combination with dabrafenib in pediatric BRAF V600-mutant low-grade glioma | 2023 | J Clin Oncol  DOI: 10.1200/JCO.22.01000 | 6 |  |
| Bouffet E, Hansford JR, Garrè ML, *et al.* | Dabrafenib plus trametinib in pediatric glioma with BRAF V600 mutations | 2023 | N Engl J Med  DOI: 10.1056/NEJMoa2303815 | 2 |  |
| Cacchione A, Fabozzi F, Carai A, *et al.* | Safety and efficacy of Mek inhibitors in the treatment of plexiform neurofibromas: a retrospective study | 2023 | Cancer Control  DOI: 10.1177/10732748221144930 | 8 |  |
| Dai YL, Choi CS, Wiltsie L, *et al.* | Selumetinib in the treatment of orbital plexiform neurofibroma: a case report | 2023 | Ophthalmic Plast Reconstr Surg  DOI: 10.1097/IOP.0000000000002330 | 9 |  |
| Fenner B, Cavazos A, Mui U, *et al.* | Drug-induced neutrophilic lobular panniculitis secondary to BRAF and MEK inhibitor used for treatment of low-grade glioma and its management | 2023 | Proc (Bayl Univ Med Cent)  DOI: 10.1080/08998280.2023.2205811 | 9 |  |
| Gross AM, Dombi E, Wolters PL, *et al.* | Long-term safety and efficacy of selumetinib in children with neurofibromatosis type 1 on a phase 1/2 trial for inoperable plexiform neurofibromas | 2023 | Neuro Oncol  DOI: 10.1093/neuonc/noad086 | 6 |  |
| Hargrave DR, Terashima K, Hara J, *et al.* | Phase II trial of dabrafenib plus trametinib in relapsed/refractory BRAF V600-mutant pediatric high-grade glioma | 2023 | J Clin Oncol  DOI: 10.1200/JCO.23.00558 | 6 |  |
| Palmeiro AG, Silva L, Pimentel B, *et al.* | MEK inhibitor-induced paronychia in a paediatric population: a tertiary centre experience | 2023 | Australas J Dermatol  DOI: 10.1111/ajd.14079 | 8 |  |
| Rush C, Sabus A, Bradley ZK, *et al.* | The incidence and characterization of weight gain associated with MEK inhibitors in pediatric patients | 2023 | Pediatr Blood Cancer  DOI: 10.1002/pbc.30182 | 8 |  |
| Suenobu S, Terashima K, Akiyama M, *et al.* | Selumetinib in Japanese pediatric patients with neurofibromatosis type 1 and symptomatic, inoperable plexiform neurofibromas: an open-label, phase I study | 2023 | Neurooncol Adv  DOI: 10.1093/noajnl/vdad054 | 6 |  |
| Whitlock JA, Geoerger B, Dunkel IJ, *et al.* | Dabrafenib, alone or in combination with trametinib, in BRAF V600-mutated pediatric Langerhans cell histiocytosis | 2023 | Blood Adv  DOI: 10.1182/bloodadvances.2022008414 | 6 |  |
| Cantor E, Meyer A, Morris SM, *et al.* | Dose-dependent seizure control with MEK inhibitor therapy for progressive glioma in a child with neurofibromatosis type 1 | 2022 | Childs Nerv Syst  DOI: 10.1007/s00381-022-05571-y | 9 |  |
| Coltin H, Perreault S, Larouche V, *et al.* | Selumetinib for symptomatic, inoperable plexiform neurofibromas in children with neurofibromatosis type 1: a national real-world case series | 2022 | Pediatr Blood Cancer  DOI: 10.1002/pbc.29633 | 8 |  |
| Crocco M, Verrico A, Milanaccio C, *et al.* | Dyslipidemia in children treated with a BRAF inhibitor for low-grade gliomas: a new side effect? | 2022 | Cancers (Basel)  DOI: 10.3390/cancers14112693 | 8 |  |
| Du Y, Hu YT. | Association of bilateral pan-uveitis with the use of trametinib for Langerhans cell histiocytosis | 2022 | Am J Transl Res  PMID: 36628254 | 9 |  |
| Eckstein OS, Allen CE, Williams PM, *et al.* | Phase II study of selumetinib in children and young adults with tumors harboring activating mitogen-activated protein kinase pathway genetic alterations: arm E of the NCI-COG pediatric MATCH trial | 2022 | J Clin Oncol  DOI: 10.1200/JCO.21.02840 | 6 |  |
| Gupta R, Craddock MF. | Successful treatment of a child with MEK inhibitor-induced acneiform eruption with low-dose isotretinoin | 2022 | Pediatr Dermatol  DOI: 10.1111/pde.14959 | 9 |  |
| Kata K, Rodriguez-Quintero JC, Arevalo OD, *et al.* | BRAF/MEK dual inhibitors therapy in progressive and anaplastic pleomorphic xanthoastrocytoma: case series and literature review | 2022 | J Natl Compr Canc Netw  DOI: 10.6004/jnccn.2022.7046 | 8 |  |
| Leclair NK, Lambert W, Roche K, *et al.* | Early experience with targeted therapy as a first-line adjuvant treatment for pediatric low-grade glioma | 2022 | Neurosurg Focus  DOI: 10.3171/2022.9.FOCUS22410 | 8 |  |
| Rosenberg T, Yeo KK, Mauguen A, *et al.* | Upfront molecular targeted therapy for the treatment of BRAF-mutant pediatric high-grade glioma | 2022 | Neuro Oncol  DOI: 10.1093/neuonc/noac096 | 8 |  |
| Salek M, Oak N, Hines M, *et al.* | Development of BRAFV600E-positive acute myeloid leukemia in a patient on long-term dabrafenib for multisystem LCH | 2022 | Blood Adv  DOI: 10.1182/bloodadvances.2021006229 | 9 |  |
| Solano-Páez P, Fonseca A, Baroni LV, *et al.* | Clinical and molecular characteristics of pediatric low-grade glioma complicated with ventriculo-peritoneal shunt related ascites | 2022 | J Neurooncol  DOI: 10.1007/s11060-022-03956-2 | 8 |  |
| Trippett T, Toledano H, Campbell Hewson Q, *et al.* | Cobimetinib in pediatric and young adult patients with relapsed or refractory solid tumors (iMATRIX-cobi): a multicenter, phase I/II study | 2022 | Target Oncol  DOI: 10.1007/s11523-022-00888-9 | 6 |  |
| Vaassen P, Dürr NR, Rosenbaum T. | Treatment of plexiform neurofibromas with MEK inhibitors: first results with a new therapeutic option | 2022 | Neuropediatrics  DOI: 10.1055/s-0041-1740549 | 8 |  |
| Volontè M, Isoletta E, Gordon S, *et al.* | Acneiform rash as a side effect of selumetinib in a child with neurofibromatosis type 1 treated for inoperable plexiform neurofibromas: good results with doxycycline | 2022 | Dermatol Ther  DOI: 10.1111/dth.15607 | 9 |  |
| Wang D, Chen XH, Wei A, *et al.* | Clinical features and treatment outcomes of pediatric Langerhans cell histiocytosis with macrophage activation syndrome-hemophagocytic lymphohistiocytosis | 2022 | Orphanet J Rare Dis  DOI: 10.1186/s13023-022-02276-y | 8 |  |
| Yao JF, Wang D, Ma HH, *et al.* | Characteristics and treatment outcomes of pediatric Langerhans cell histiocytosis with thymic involvement | 2022 | J Pediatr  DOI: 10.1016/j.jpeds.2022.01.007 | 8 |  |
| Baldo F, Magnolato A, Barbi E, *et al.* | Selumetinib side effects in children treated for plexiform neurofibromas: first case reports of peripheral edema and hair color change | 2021 | BMC Pediatr  DOI: 10.1186/s12887-021-02530-5 | 9 |  |
| Boull CL, Gardeen S, Abdali T, *et al.* | Cutaneous reactions in children treated with MEK inhibitors, BRAF inhibitors, or combination therapy: A multicenter study | 2021 | J Am Acad Dermatol  DOI: 10.1016/j.jaad.2020.07.044 | 8 |  |
| Dávila Osorio VL, Vicente MA, Baselga E, *et al.* | Adverse cutaneous effects of mitogen-activated protein kinase inhibitors in children | 2021 | Pediatr Dermatol  DOI: 10.1111/pde.14354 | 8 |  |
| Deitch-Harel I, Raskin E, Habot-Wilner Z, *et al.* | Uveitis induced by biological agents used in cancer therapy | 2021 | Ocul Immunol Inflamm  DOI: 10.1080/09273948.2020.1760310 | 8 |  |
| Fangusaro J, Onar-Thomas A, Poussaint TY, *et al.* | A phase II trial of selumetinib in children with recurrent optic pathway and hypothalamic low-grade glioma without NF1: a Pediatric Brain Tumor Consortium study | 2021 | Neuro Oncol  DOI: 10.1093/neuonc/noab047 | 6 |  |
| Lazow MA, Lawson SA, Salloum R, *et al.* | Trametinib-associated hyponatremia in a child with low-grade glioma is not seen following treatment with alternative MEK inhibitor | 2021 | J Pediatr Hematol Oncol  DOI: 10.1097/MPH.0000000000001859 | 9 |  |
| Ollech A, Yalon M, Abebe-Campino G, *et al.* | Cutaneous adverse events to targeted therapies and immuno-therapies in children: a retrospective study of 103 patients from two tertiary haemato-oncology referral centres | 2021 | Acta Derm Venereol  DOI: 10.2340/00015555-3867 | 8 |  |
| Pérez JPM, Muchart J, López VS, *et al.* | Targeted therapy for pediatric low-grade glioma | 2021 | Childs Nerv Syst  DOI: 10.1007/s00381-021-05138-3 | 8 |  |
| Ronsley R, Hounjet CD, Cheng S, *et al.* | Trametinib therapy for children with neurofibromatosis type 1 and life-threatening plexiform neurofibroma or treatment-refractory low-grade glioma | 2021 | Cancer Med  DOI: 10.1002/cam4.3910 | 8 |  |
| Sandor KP, Grossniklaus HE, Lenhart PD, *et al.* | Association of conjunctival plaques with BRAF inhibitor treatment in patients with pediatric brain tumor | 2021 | JAMA Ophthalmol  DOI: 10.1001/jamaophthalmol.2021.0169 | 9 |  |
| Tardieu M, Néron A, Duvert-Lehembre S, *et al.* | Cutaneous adverse events in children treated with vemurafenib for refractory BRAF(V600E) mutated Langerhans cell histiocytosis | 2021 | Pediatr Blood Cancer  DOI: 10.1002/pbc.29140 | 8 |  |
| Toledano H, Dotan G, Friedland R, *et al.* | Trametinib for orbital plexiform neurofibromas in young children with neurofibromatosis type 1 | 2021 | Childs Nerv Syst  DOI: 10.1007/s00381-021-05127-6 | 8 |  |
| Yang Y, Wang D, Cui L, *et al.* | Effectiveness and safety of dabrafenib in the treatment of 20 Chinese children with BRAFV600E-mutated Langerhans cell histiocytosis | 2021 | Cancer Res Treat  DOI: 10.4143/crt.2020.769 | 8 |  |
| Baldo F, Grasso AG, Cortellazzo Wiel L, *et al.* | Selumetinib in the treatment of symptomatic intractable plexiform neurofibromas in neurofibromatosis type 1: a prospective case series with emphasis on side effects | 2020 | Paediatr Drugs  DOI: 10.1007/s40272-020-00399-y | 8 |  |
| Egan G, Hamilton J, McKeown T, *et al.* | Trametinib toxicities in patients with low-grade gliomas and diabetes insipidus: related findings? | 2020 | J Pediatr Hematol Oncol  DOI: 10.1097/MPH.0000000000001427 | 9 |  |
| Espírito Santo V, Passos J, *et al.* | Selumetinib for plexiform neurofibromas in neurofibromatosis type 1: a single-institution experience | 2020 | J Neurooncol  DOI: 10.1007/s11060-020-03443-6 | 8 |  |
| Gorsi H, Marupudi NI, Sood S, *et al.* | Pneumocephalus in a pediatric patient with glioma receiving trametinib | 2020 | Pediatr Neurosurg  DOI: 10.1159/000503639 | 9 |  |
| Gross AM, Wolters PL, Dombi E, *et al.* | Selumetinib in children with inoperable plexiform neurofibromas | 2020 | N Engl J Med  DOI: 10.1056/NEJMoa1912735 | 6 |  |
| Manoharan N, Choi J, Chordas C, *et al.* | Trametinib for the treatment of recurrent/progressive pediatric low-grade glioma | 2020 | J Neurooncol  DOI: 10.1007/s11060-020-03592-8 | 8 |  |
| Martínez-de-Espronceda I, Bernabeu-Wittel J, Azcona M, *et al.* | Recalcitrant trametinib-induced paronychia treated successfully with topical timolol in a pediatric patient | 2020 | Dermatol Ther  DOI: 10.1111/dth.13164 | 9 |  |
| Messinger YH, Bostrom BC, Olson DR, *et al.* | Langerhans cell histiocytosis with BRAF p.N486_P490del or MAP2K1 p.K57_G61del treated by the MEK inhibitor trametinib | 2020 | Pediatr Blood Cancer  DOI: 10.1002/pbc.28712 | 9 |  |
| Nicolaides T, Nazemi KJ, Crawford J, *et al.* | Phase I study of vemurafenib in children with recurrent or progressive BRAF(V600E) mutant brain tumors: Pacific Pediatric Neuro-Oncology Consortium study (PNOC-002) | 2020 | Oncotarget  DOI: 10.18632/oncotarget.27600 | 6 |  |
| Paul MR, Pehlivan KC, Milburn M, *et al.* | Trametinib-based treatment of pediatric CNS tumors: a single institutional experience | 2020 | J Pediatr Hematol Oncol  DOI: 10.1097/MPH.0000000000001819 | 8 |  |
| Peterson RK, McKeown T, Tabori U, *et al.* | Neuropsychological impact of trametinib in pediatric low-grade glioma: a case series | 2020 | Pediatr Blood Cancer  DOI: 10.1002/pbc.28690 | 8 |  |
| Selt F, van Tilburg CM, Bison B, *et al.* | Response to trametinib treatment in progressive pediatric low-grade glioma patients | 2020 | J Neurooncol  DOI: 10.1007/s11060-020-03640-3 | 8 |  |
| Sun Q, Antaya RJ. | Treatment of MEK inhibitor-induced paronychia with doxycycline | 2020 | Pediatr Dermatol  DOI: 10.1111/pde.14276 | 9 |  |
| Donadieu J, Larabi IA, Tardieu M, *et al.* | Vemurafenib for refractory multisystem Langerhans cell histiocytosis in children: an international observational study | 2019 | J Clin Oncol  DOI: 10.1200/JCO.19.00456 | 8 |  |
| Fangusaro J, Onar-Thomas A, Young Poussaint T, *et al.* | Selumetinib in paediatric patients with BRAF-aberrant or neurofibromatosis type 1-associated recurrent, refractory, or progressive low-grade glioma: a multicentre, phase 2 trial | 2019 | Lancet Oncol  DOI: 10.1016/S1470-2045(19)30277-3 | 6 |  |
| Hargrave DR, Bouffet E, Tabori U, *et al.* | Efficacy and safety of dabrafenib in pediatric patients with BRAF V600 mutation-positive relapsed or refractory low-grade glioma: results from a phase I/IIa study | 2019 | Clin Cancer Res  DOI: 10.1158/1078-0432.CCR-19-2177 | 6 |  |
| Jew OS, Provini LE, Treat JR. | Severe vemurafenib-induced photosensitivity in a 6-year-old boy | 2019 | Pediatr Dermatol  DOI: 10.1111/pde.13720 | 9 |  |
| Kieran MW, Geoerger B, Dunkel IJ, *et al.* | A phase I and pharmacokinetic study of oral dabrafenib in children and adolescent patients with recurrent or refractory BRAF V600 mutation-positive solid tumors | 2019 | Clin Cancer Res  DOI: 10.1158/1078-0432.CCR-17-3572 | 6 |  |
| Petruzzellis G, Valentini D, Del Bufalo F, *et al.* | Vemurafenib treatment of pleomorphic xanthoastrocytoma in a child with Down syndrome | 2019 | Front Oncol  DOI: 10.3389/fonc.2019.00277 | 9 |  |
| Song H, Zhong CS, Kieran MW, *et al.* | Cutaneous reactions to targeted therapies in children with CNS tumors: a cross-sectional study | 2019 | Pediatr Blood Cancer  DOI: 10.1002/pbc.27682 | 8 |  |
| Amayiri N, Swaidan M, Al-Hussaini M, *et al.* | Sustained response to targeted therapy in a patient with disseminated anaplastic pleomorphic xanthoastrocytoma | 2018 | J Pediatr Hematol Oncol  DOI: 10.1097/MPH.0000000000001032 | 9 |  |
| Chen L, Hsi AC, Kothari A, *et al.* | Granulomatous dermatitis secondary to vemurafenib in a child with Langerhans cell histiocytosis | 2018 | Pediatr Dermatol  DOI: 10.1111/pde.13613 | 9 |  |
| Del Bufalo F, Ceglie G, Cacchione A, et al. | BRAF V600E inhibitor (Vemurafenib) for BRAF V600E mutated low grade gliomas | 2018 | Front Oncol  DOI: 10.3389/fonc.2018.00526 | 8 |  |
| Kondyli M, Larouche V, Saint-Martin C, *et al.* | Trametinib for progressive pediatric low-grade gliomas | 2018 | J Neurooncol  DOI: 10.1007/s11060-018-2971-9 | 8 |  |
| Marks AM, Bindra RS, DiLuna ML, *et al.* | Response to the BRAF/MEK inhibitors dabrafenib/trametinib in an adolescent with a BRAF V600E mutated anaplastic ganglioglioma intolerant to vemurafenib | 2018 | Pediatr Blood Cancer  DOI: 10.1002/pbc.26969 | 9 |  |
| Schreck KC, Guajardo A, Lin DDM, *et al.* | Concurrent BRAF/MEK inhibitors in BRAF V600-mutant high-grade primary brain tumors | 2018 | J Natl Compr Canc Netw.  DOI: 10.6004/jnccn.2017.7052 | 9 |  |
| Upadhyaya SA, Robinson GW, Harreld JH, *et al.* | Marked functional recovery and imaging response of refractory optic pathway glioma to BRAFV600E inhibitor therapy: a report of two cases | 2018 | Childs Nerv Syst  DOI: 10.1007/s00381-018-3739-4 | 9 |  |
| Banerjee A, Jakacki RI, Onar-Thomas A, *et al.* | A phase I trial of the MEK inhibitor selumetinib (AZD6244) in pediatric patients with recurrent or refractory low-grade glioma: a Pediatric Brain Tumor Consortium (PBTC) study | 2017 | Neuro Oncol  DOI: 10.1093/neuonc/now282 | 6 |  |
| Boull C, Hook K, Moertel C, *et al.* | Cutaneous reactions in children treated with the mitogen-activated protein kinase extracellular signal-regulated kinase inhibitor trametinib for neural tumors | 2017 | Pediatr Dermatol  DOI: 10.1111/pde.13038 | 8 |  |
| Drobysheva A, Klesse LJ, Bowers DC, *et al.* | Targeted MAPK pathway inhibitors in patients with disseminated pilocytic astrocytomas | 2017 | J Natl Compr Canc Netw  DOI: 10.6004/jnccn.2017.0139 | 8 |  |
| Finelt N, Lulla RR, Melin-Aldana H, *et al.* | Bumps in the road: panniculitis in children and adolescents treated with vemurafenib | 2017 | Pediatr Dermatol  DOI: 10.1111/pde.13148 | 9 |  |
| Kinsler VA, O'Hare P, Jacques T, Hargrave D, Slater O. | MEK inhibition appears to improve symptom control in primary NRAS-driven CNS melanoma in children | 2017 | Br J Cancer  DOI: 10.1038/bjc.2017.49 | 9 |  |
| Aguilera D, Janss A, Mazewski C, *et al.* | Successful retreatment of a child with a refractory brainstem ganglioglioma with vemurafenib | 2016 | Pediatr Blood Cancer  DOI: 10.1002/pbc.25787 | 9 |  |
| Avery RA, Trimboli-Heidler C, *et al.* | Separation of outer retinal layers secondary to selumetinib | 2016 | J AAPOS  DOI: 10.1016/j.jaapos.2016.01.012 | 9 |  |
| Dombi E, Baldwin A, Marcus LJ, *et al.* | Activity of selumetinib in neurofibromatosis type 1-related plexiform neurofibromas | 2016 | N Engl J Med  DOI: 10.1056/NEJMoa1605943 | 6 |  |
| Modak S, Asante-Korang A, Steinherz LJ, *et al.* | Trametinib-induced left ventricular dysfunction in a child with relapsed neuroblastoma | 2015 | J Pediatr Hematol Oncol  DOI: 10.1097/MPH.0000000000000364 | 9 |  |
| West ES, Williams VL, Morelli JG. | Vemurafenib-induced neutrophilic panniculitis in a child with a brainstem glioma | 2015 | Pediatr Dermatol  DOI: 10.1111/pde.12316 | 9 |  |
| Bautista F, Paci A, Minard-Colin V, *et al.* | Vemurafenib in pediatric patients with BRAFV600E mutated high-grade gliomas | 2014 | Pediatr Blood Cancer  DOI: 10.1002/pbc.24891 | 9 |  |
| **Journal Articles: Systematic Reviews with Meta-Analyses** | | | | | |
| Han Y, Li B, Yu X, *et al.* | Efficacy and safety of selumetinib in patients with neurofibromatosis type 1 and inoperable plexiform neurofibromas: a systematic review and meta-analysis | 2024 | J Neurol  DOI: 10.1007/s00415-024-12301-8 | 6 |  |
| Mohapatra D, Gupta AK, Haldar P, *et al.* | Efficacy and safety of vemurafenib in Langerhans cell histiocytosis (LCH): a systematic review and meta-analysis | 2023 | Pediatr Hematol Oncol  DOI: 10.1080/08880018.2022.2072986 | 8 |  |
| Hwang J, Yoon HM, Lee BH, *et al.* | Efficacy and safety of selumetinib in pediatric patients with neurofibromatosis type 1: a systematic review and meta-analysis | 2022 | Neurology  DOI: 10.1212/WNL.0000000000013296 | 6 |  |
| Sharawat IK, Panda PK, Sihag RK, *et al.* | Efficacy and safety profile of selumetinib in symptomatic inoperable plexiform neurofibromas | 2022 | J Neurosurg Sci  DOI: 10.23736/S0390-5616.21.05528-4 | 6 |  |
| Wang D, Ge LL, Guo ZZ, *et al.* | Efficacy and safety of trametinib in neurofibromatosis type 1-associated plexiform neurofibroma and low-grade glioma: a systematic review and meta-analysis | 2022 | Pharmaceuticals (Basel)  DOI: 10.3390/ph15080956 | 6 |  |
| **Journal Articles: Systematic Reviews Without Meta-Analyses** | | | | | |
| Fisher B, Meyer A, Brown A, *et al.* | Evidence-based recommendations for education provided to patients and families regarding the adverse events of ALK and MEK inhibitors: a systematic review from the Children's Oncology Group | 2024 | J Pediatr Hematol Oncol Nurs  DOI: 10.1177/27527530231206101 | N/A |  |
| Suresh KV, Xu AL, Groves ML, *et al.* | Spinal screening, malignancy, medical therapy, and surgical correction of deformity in pediatric patients with neurofibromatosis type 1: a systematic review | 2022 | J Pediatr Orthop B  DOI: 10.1097/BPB.0000000000000961 | N/A |  |
| **Journal Articles: Narrative Reviews** | | | | | |
| El Khoury JV, Wehbe S, Attieh F, *et al.* | A critical review of RAF inhibitors in BRAF-mutated glioma treatment | 2024 | Pharmacogenomics  DOI: 10.1080/14622416.2024.2355859 | N/A |  |
| Dar MS, Shahid N, Waqas A, *et al.* | Dabrafenib plus trametinib: a breakthrough in pediatric low-grade glioma therapy | 2024 | Health Sci Rep  DOI: 10.1002/hsr2.1841 | N/A |  |
| Dhillon S. | Tovorafenib: first approval | 2024 | Drugs  DOI: 10.1007/s40265-024-02069-6 | N/A |  |
| Hanrahan AJ, Chen Z, Rosen N, *et al.* | BRAF - a tumour-agnostic drug target with lineage-specific dependencies | 2024 | Nat Rev Clin Oncol  DOI: 10.1038/s41571-023-00852-0 | N/A |  |
| Abla O. | Langerhans cell histiocytosis: promises and caveats of targeted therapies in high-risk and CNS disease | 2023 | Hematology Am Soc Hematol Educ Program  DOI: 10.1182/hematology.2023000439 | N/A |  |
| Gouda MA, Subbiah V. | Expanding the benefit: dabrafenib/trametinib as tissue-agnostic therapy for BRAF V600E-positive adult and pediatric solid tumors | 2023 | Am Soc Clin Oncol Educ Book  DOI: 10.1200/EDBK_404770 | N/A |  |
| Trinder SM, McKay C, Power P, *et al.* | BRAF-mediated brain tumors in adults and children: A review and the Australian and New Zealand experience | 2023 | Front Oncol  DOI: 10.3389/fonc.2023.1154246 | N/A |  |
| Acar S, Armstrong AE, *et al.* | Plexiform neurofibroma: shedding light on the investigational agents in clinical trials | 2022 | Expert Opin Investig Drugs  DOI: 10.1080/13543784.2022.2022120 | N/A |  |
| Anderson MK, Johnson M, Thornburg L, *et al.* | A review of selumetinib in the treatment of neurofibromatosis type 1-related plexiform neurofibromas | 2022 | Ann Pharmacother  DOI: 10.1177/10600280211046298 | N/A |  |
| Murugappan MN, King-Kallimanis BL, Reaman GH, *et al.* | Patient-reported outcomes in pediatric cancer registration trials: a US Food and Drug Administration perspective | 2022 | J Natl Cancer Inst  DOI: 10.1093/jnci/djab087 | N/A |  |
| Campagne O, Yeo KK, Fangusaro J, *et al.* | Clinical pharmacokinetics and pharmacodynamics of selumetinib | 2021 | Clin Pharmacokinet  DOI: 10.1007/s40262-020-00967-y | N/A |  |
| Peeters SM, Muftuoglu Y, Na B, *et al.* | Pediatric gliomas: molecular landscape and emerging targets | 2021 | Neurosurg Clin N Am  DOI: 10.1016/j.nec.2020.12.001 | N/A |  |
| Solares I, Viñal D, Morales-Conejo M, *et al.* | Novel molecular targeted therapies for patients with neurofibromatosis type 1 with inoperable plexiform neurofibromas: a comprehensive review | 2021 | ESMO Open  DOI: 10.1016/j.esmoop.2021.100223 | N/A |  |
| Klesse LJ, Jordan JT, Radtke HB, *et al.* | The use of MEK inhibitors in neurofibromatosis type 1-associated tumors and management of toxicities | 2020 | Oncologist  DOI: 10.1634/theoncologist.2020-0069 | N/A |  |
| Mueller T, Stucklin ASG, Postlmayr A, *et al.* | Advances in targeted therapies for pediatric brain tumors | 2020 | Curr Treat Option Ne  DOI: 10.1007/s11940-020-00651-3 | N/A |  |
| Maraka S, Janku F. | BRAF alterations in primary brain tumors | 2018 | Discov Med  PMID: 30265855 | N/A |  |
| Rizzo D, Ruggiero A, Amato M, *et al.* | BRAF and MEK inhibitors in pediatric glioma: new therapeutic strategies, new toxicities | 2016 | Expert Opin Drug Metab Toxicol  DOI: 10.1080/17425255.2016.1214710 | N/A |  |
| **Journal Articles: Approval Summaries and Other Articles** | | | | | |
| Barbato MI, Nashed J, Bradford D, *et al.* | FDA approval summary: dabrafenib in combination with trametinib for BRAFV600E mutation-positive low-grade glioma | 2024 | Clin Cancer Res  DOI: 10.1158/1078-0432.CCR-23-1503 | N/A |  |
| Pearson AD, Allen C, Fangusaro J, *et al.* | Paediatric strategy forum for medicinal product development in mitogen-activated protein kinase pathway inhibitors: ACCELERATE in collaboration with the European Medicines Agency with participation of the Food and Drug Administration | 2022 | Eur J Cancer  DOI: 10.1016/j.ejca.2022.09.036 | N/A |  |
| Casey D, Demko S, Sinha A, *et al.* | FDA approval summary: selumetinib for plexiform neurofibroma | 2021 | Clin Cancer Res  DOI: 10.1158/1078-0432.CCR-20-5032 | N/A |  |
| Burki TK. | Selumetinib for children with plexiform neurofibromas | 2017 | Lancet Oncol  DOI: 10.1016/S1470-2045(17)30009-8 | N/A |  |
| **Congress Abstracts/Posters** | | | | | |
| De La Fuente MI, Rodon Ahnert J, Yaeger R, *et al.* | Abstract no. 3006. Safety and efficacy of the novel BRAF inhibitor FORE8394 in patients with advanced solid and CNS tumors: results from a phase 1/2a study | 2023 | ASCO | 6 |  |
| Hemenway M | Abstract no. 52. Neurofibromatosis therapeutics program: program development, tumor treatment, and side effect management | 2023 | Global NF-1 | N/A |  |
| Khuong-Quang D-A, Nysom K, Landi D, *et al.* | Abstract no. CTNI-37. Clinical activity of RAF inhibitor tovorafenib according to prior MAPK inhibitor treatment in the registrational pediatric low-grade glioma arm of the phase 2 FIREFLY-1 (PNOC026) study | 2023 | SNO | 6 |  |
| Kiaei DS, Larouche V, Décarie J-C, Tabori U, *et al.* | TRAM-01: A phase 2 study of trametinib for pediatric patients with neurofibromatosis type 1 and plexiform neurofibromas | 2023 | Global NF-1 | 6 |  |
| Kilburn LB, Khuong-Quang D-A, Nysom K, *et al.* | Abstract no. 10004. Clinical activity of pan-RAF inhibitor tovorafenib in the registrational pediatric low-grade glioma arm of the phase 2 FIREFLY-1 (PNOC026) study | 2023 | ASCO | 6 |  |
| Kim H, Yoon HM, Kim EK, *et al.* | Abstract no. 70. Diverse clinical effects of selumetinib in Korean children and adults with neurofibromatosis type I | 2023 | Global NF-1 | 6 |  |
| Klein A, Moorman B, Huang M | Abstract no. 271. Severe gastrointestinal bleeding secondary to trametinib in pediatric low-grade glioma | 2023 | ASPHO | 9 |  |
| Lazow M, Thomas D, Cottrell C, *et al.* | Abstract no. LGG-14. Treatment of two pediatric FGFR-altered low-grade glioneuronal tumors with MEK inhibition | 2023 | SNO Peds | 9 |  |
| Li W, Mao Y, Kang Z, *et al.* | Abstract no. 499O. A phase II study to explore the efficacy and safety of FCN-159 in recurrent or progressive pediatric low-grade glioma (pLGG) with MAPK pathway-activated | 2023 | ESMO | 6 |  |
| Liang C, Vandenheuvel J, Allen S | Abstract no. 185. A case of severe cystic lung disease in risk organ positive multisystem LCH | 2023 | ASPHO | 9 |  |
| McThenia S, Reddy K, Goldman-Yassen AE, *et al.* | Abstract no. LGG-23. Outcomes utilizing BRAF inhibition monotherapy in BRAF^V600E^ mutated pediatric low grade gliomas | 2023 | SNO Peds | 8 |  |
| Nysom K, Kilburn L, Leary S, *et al.* | Abstract no. CTNI-24. Clinical activity and safety of the RAF inhibitor tovorafenib in patients with optic pathway gliomas in the registrational pediatric low-grade glioma arm of the phase 2 FIREFLY-1 (PNOC026) study | 2023 | SNO | 6 |  |
| Perreault S | A phase 2 study of trametinib for patients with pediatric low grade glioma with activation of the MAPK/ERK pathway | 2023 | SIOP | 6 |  |
| Reddy AT, Fisher MJ, Dombi E, *et al.* | Abstract no. 110. Durability of binimetinib response and retreatment in pediatric and adult patients with neurofibromatosis type 1 associated plexiform neurofibromas: a report from the NFCTC and PNOC | 2023 | Global NF-1 | 6 |  |
| Rosenbaum T, Reinhard Dürr N, Vaassen P | Abstract no. 122. MEK inhibition in NF1-associated plexiform neurofibromas: 5-years experience in a tertiary treatment center in Germany | 2023 | Global NF-1 | 8 |  |
| Sumerauer D, Trkova K, Koblizek M, *et al.* | Abstract no. LGG-07. Targeted therapy in pediatric low-grade gliomas; real world data in the context of conventional treatment modalities | 2023 | SNO Peds | 8 |  |
| Viskochil D, Wysocki M, Learoyd M, *et al.* | Abstract no. 132. A phase 1 study to assess the effect of food on the pharmacokinetics (PK) and gastrointestinal (GI)  tolerability of selumetinib in adolescents with neurofibromatosis type 1 (NF1)-related plexiform  neurofibromas (PN) | 2023 | Global NF-1 | 6 |  |
| Allmon P, Delaney A, Bourque MS, *et al.* | Abstract no. 501. Management of refractory hyperandrogenism with BRAF V600E inhibition in a patient with ganglioglioma | 2022 | ASPHO | 9 |  |
| Apps J, Peet A, English M, *et al.* | Abstract no. OTHR-20. Precision neuro-oncology in the real world. Opportunity and challenges from a UK Oncology Centre | 2022 | ISPNO | 8 |  |
| Bahng HH, Malvar J, Chi Y-Y, *et al.* | Abstract no. LGG-62. Weight change in pediatric patients treated with MEK inhibitors: a retrospective cohort study | 2022 | ISPNO | 8 |  |
| Bendel A, Skrypek M, Johnson H, *et al.* | Abstract no. LGG-63. MEK inhibitor-associated retinopathy (MEKAR) in a pediatric patient with an optic pathway glioma | 2022 | ISPNO | 9 |  |
| Bregonje S, de Vos-Kerkhof E, Engels FK, *et al.* | Poster. Effectivity and toxicity of off-label treatment with trametinib monotherapy or in combination with dabrafenib in children with relapsed or refractory brain tumor | 2022 | ISPNO | 8 |  |
| Cantor E, Meyer A, Ogle A, *et al.* | Abstract no. LGG-38. Dose-dependent seizure control for an NF1 patient treated via MEK-inhibition for optic pathway glioma | 2022 | ISPNO | 9 |  |
| Cantor E, Shatara M, Meyers A, *et al.* | Abstract no. LGG-02. Cardiac toxicity in patients receiving single-agent MEK inhibition | 2022 | ISPNO | 8 |  |
| Chan P, Sabus A, Hemenway M, *et al.* | Abstract no. LGG-42. Thromboembolic toxicity observed with concurrent trametinib and lenalidomide therapy | 2022 | ISPNO | 8 |  |
| Fangusaro J, Onar-Thomas A, Young Poussaint T, *et al.* | Abstract no. LGG-06. Selumetinib in pediatric patients with non-neurofibromatosis type 1-associated, non-optic pathway (OPG) and non-pilocytic recurrent/progressive low-grade glioma harboring BRAFV600E mutation or BRAF-KIAA1549 fusion: a multicenter prospective Pediatric Brain Tumor Consortium (PBTC) Phase 2 trial | 2022 | ISPNO | 6 |  |
| Gluck M, Ben-Amitai D, Friedland R, *et al.* | Abstract no. OTHR-37. Pediatrics cutaneous reactions in patient treated with the mitogen-activated protein kinase extracellular signal-regulated kinase inhibitor trametinib | 2022 | ISPNO | 8 |  |
| Hoffman LM, Mulcahy Levy J, Kilburn L, *et al.* | Abstract no. EPCT-01. Pediatric Brain Tumor Consortium (PBTC)-055: a phase I study of trametinib and hydroxychloroquine (HCQ) for BRAF-fusion or neurofibromatosis type-1 (NF1)-associated pediatric gliomas | 2022 | ISPNO | 6 |  |
| Kiaei DS, Larouche V, Décarie J-C, *et al.* | Abstract no. NFB-08. TRAM-01: a phase 2 study of trametinib for pediatric patients with neurofibromatosis type 1 and plexiform neurofibromas | 2022 | SNO | 6 |  |
| Kilburn L, Landi D, Leary S, *et al.* | Abstract no. CTNI-68. FIREFLY-1 (PNOC026): phase 2 study of pan-RAF inhibitor tovorafenib in pediatric and young adult patients with RAF-altered recurrent or progressive low-grade glioma or advanced solid tumors | 2022 | SNO | 6 |  |
| Meyer A, Armstrong A, Cluster A, *et al.* | Abstract no. OTHR-18. A pilot study evaluating the access, utilization, and patient satisfaction of basic skin care products among pediatric patients prescribed medications that target the mitogen-activated protein kinase pathway | 2022 | ISPNO | 6 |  |
| Meyer A, Ogle A, Shatara M, *et al.* | Abstract (and poster) no. OTHR-09. The prevalence and complex management of MEK inhibitor induced cutaneous side effects | 2022 | ISPNO | 8 |  |
| Papusha L | Infant low-grade gliomas: molecular characteristics and results of targeted therapy | 2022 | SIOP | 8 |  |
| Rangan K, Bira K, Bryant L, *et al.* | Abstract no. NURS-01. Development and implementation of a MEK inhibitor targeted therapy standardized educational curriculum for pediatric oncology patients, caregivers, and clinical staff | 2022 | ISPNO | N/A |  |
| Robison N, Pauly J, Malvar J, *et al.* | Abstract no. LTBK-04. MEK162 (binimetinib) in children with progressive or recurrent low-grade glioma: a multi-institutional phase II and target validation study | 2022 | ISPNO | 6 |  |
| Rosenberg T, Yeo KK, Mauguen A, *et al.* | Abstract no. HGG-34. Upfront molecular targeted therapy for the treatment of BRAF-mutant pediatric high-grade glioma | 2022 | ISPNO | 8 |  |
| Verrico A, Crocco M, La Porta E, *et al.* | Abstract no. LGG-34. Nephrological impact of BRAF inhibitors in a pediatric population of central nervous system tumors: a single institution experience | 2022 | ISPNO | 8 |  |
| Vinitsky A, Chiang J, Bag AK, *et al.* | Abstract no. SJ901. Phase I/II evaluation of single agent mirdametinib (PD-0325901), a brain-penetrant MEK1/2 inhibitor, for the treatment of children, adolescents, and young adults with low-grade glioma (LGG) | 2022 | ISPNO | 6 |  |
| Wright K, Kline C, Abdelbaki M, *et al.* | Abstract no. CTNI-53. PNOC014: Phase IB study results of Day101 (tovorafenib) for children with low-grade gliomas (LGGs) and other RAS/RAF/MEK/ERK pathway-activated tumors | 2022 | SNO | 6 |  |
| Allen CE, Eckstein O, Williams PM, *et al.* | Selumetinib in patients with tumors with MAPK pathway alterations: Results from Arm E of the NCI-COG pediatric MATCH trial. | 2021 | ASCO | 6 |  |
| Green S, Walter A, Piatt J, *et al.* | Abstract no. LGG-12. Safety and efficacy of dual therapy with dabrafenib and trametinib in an infant with BRAF V600E mutant inoperable low grade glioma | 2021 | SNO Peds | 9 |  |
| Ku DTL, Liu APY, Fu E, *et al.* | Abstract no. LGG-17. Clinical outcome of pediatric low grade glioma with positive BRAF-fusion treated with MEK inhibitor | 2021 | SNO Peds | 8 |  |
| Rosenberg T, Yeo KK, Joshirao M, *et al.* | Abstract no. HGG-37. Upfront targeted therapy for the treatment of BRAFV600E-mutant pediatric high-grade glioma – a multi-institutional experience | 2021 | SNO Peds | 8 |  |
| Tsai J, Vogelzang J, Sousa C, *et al.* | Abstract no. LGG-03. Long-term follow up of targeted therapy in pediatric low-grade gliomas: the DANA-FARBER/Boston children’s experience | 2021 | SNO Peds | 8 |  |
| Barbour M, Huang MA | Abstract no. LGG-12. Trametinib for pediatric low grade gliomas: a single institution experience | 2020 | ISPNO | 8 |  |
| Bardi Lola G, Sato M | Abstract no. LGG-11. Institutional experience of BRAF targeting therapy | 2020 | ISPNO | 8 |  |
| Bouffet E, Whitlock JA, Moertel C, *et al.* | Abstract no. LGG-49. Safety and efficacy of trametinib (T) monotherapy and dabrafenib + trametinib (D+T) combination therapy in pediatric patients with BRAFV600-mutant low-grade glioma (LGG) | 2020 | ISPNO | 6 |  |
| Hounjet CD, Ronsley R, Cheng S, *et al.* | Abstract no. NFB-12. Trametinib therapy for pediatric patients with refractory low grade glioma or extensive symptomatic plexiform neurofibroma | 2020 | ISPNO | 9 |  |
| Lazow MA, Lawson SA, Salloum R, *et al.* | Abstract no. LGG-30. Trametinib-associated hyponatremia in a child with low grade glioma is not seen following treatment with alternative MEK inhibitor | 2020 | ISPNO | 9 |  |
| McKeown T, Lara-Corrales I, Cote A | Abstract no. NURS-04. Combination of neuro-oncology and dermatology clinics improve the management and knowledge of skin-related toxicities with MEK and BRAF targeted therapy | 2020 | ISPNO | N/A |  |
| Mueller S, Reddy AT, Dombi E, *et al.* | Abstract no. NFB-17. MEK inhibitor binimetinib shows clinical activity in children with neurofibromatosis type 1-associated plexiform neurofibromas: a report from PNOC and the NF Clinical Trials Consortium | 2020 | ISPNO | 6 |  |
| Nguyen T, McMahon K, Hemenway M, *et al.* | Abstract no. LGG-27. Targeted therapy for pediatric low-grade gliomas and plexiform neurofibromas with trametinib | 2020 | ISPNO | 8 |  |
| Robison N, Pauly J, Malvar M, *et al.* | Abstract no. LGG-52. Binimetinib in children with progressive or recurrent low-grade glioma not associated with neurofibromatosis type 1: initial results from a multi-institutional phase II study | 2020 | ISPNO | 6 |  |
| Sato A, Millard N, Perez F, *et al.* | Abstract no. NFB-13. Trametinib for plexiform neurofibroma and recurrent low-grade glioma | 2020 | ISPNO | 8 |  |
| Toledano H, Dotan G, Friedland R, *et al.* | Abstract no. NFB-03. Trametinib for orbital plexiform neurofibromas in young children with NF1 | 2020 | ISPNO | 8 |  |
| Winzent S, Sabus A, Hemenway M, *et al.* | Abstract no. MODL-01. Safety in concomitant use of MEK and BRAF inhibitors with bevacizumab | 2020 | ISPNO | 8 |  |
| Wright K, Krzykwa E, Greenspan L, *et al.* | Abstract no. EPCT-01. Phase I study of Day101 (TAK580) in children and young adults with radiographically recurrent or progressive low-grade glioma (LGG) | 2020 | ISPNO | 6 |  |
| Tanaka R, Khatua S, Zaky W, *et al.* | Abstract no. EPT-17. Phase I study of BRAF inhibitor (vemurafenib) in combination with the mtor inhibitor (everolimus) in BRAF^V600E^ positive childhood and aya brain tumors | 2016 | Neuro Oncol  DOI: [10.1093/neuonc/now069.16](https://doi.org/10.1093/neuonc/now069.16) | 6 |  |

### **Supplementary Table 9.** Statements on the Incidence, Severity and Management of AEs Occurring in Pediatric Patients Being Treated with MAPKi Therapies for which Consensus-Level Agreement was Not Achieved

| Statement | Level of Agreement | Rationale |
| --- | --- | --- |
| *Incidence and Management of AEs* |  |  |
| Dose adjustments for most MAPKi associated AEs are still being established due to limited information available | 71% | Conflicting opinions on the understanding of how to modify MAPKi dose to address AEs |
| Available literature does not support there being significant differences in the AE profile between drugs within a single MAPKi class (e.g., between different MEKi) | 60% | Drugs within the same class can have different tolerability profiles |
| *Cutaneous AEs* |  |  |
| Eruptive nevi are a common cutaneous reaction with type I BRAFi therapies | 50% | Reportedly low incidence observed in pediatric populations |
| Premature discontinuation of MAPKi therapy is common in patients who experience cutaneous reactions | 41% | Not considered common, although more frequent in adolescents; effective AE management can reduce the likelihood of discontinuation |
| Outside of clinical trials, the CTCAE grading system is a clinically useful tool to guide management of skin AEs | 34% | CTCAE not used in clinical practice and does not account for AE impact on QoL |
| Dose reduction of MAPKi therapy is common in patients who experience cutaneous reactions | 60% | Severity of AEs and how well they can be managed are important factors to consider before dose reduction |
| Management of treatment-associated cutaneous AEs is well established | 41% | Not considered well established due to a lack of guidance or recommendations |
| Eczematous reactions are more common in pre-pubertal patients compared with post-pubertal patients receiving MAPKi therapy | 48% | A history of eczema may be a stronger predictor than patients’ developmental stage |
| *General Skin Care Management* |  |  |
| Referral to a dermatologist with experience managing patients treated with MAPKi therapy, if available, is encouraged for baseline exam/counselling prior to MAPKi initiation | 56% | Dermatology referral may not be needed in instances where oncology has measures in place to prevent and manage cutaneous AEs |
| Oral antibiotics can be used preemptively in patients with preexisting acne upon initiation of MAPKi therapy | 48% | Oral antibiotics may be used for treatment rather than prevention to avoid unnecessary antibiotic use |
| If available, psychosocial support should be suggested where needed to reduce compulsive behavior (such as obsessive or compulsive scratching or nail biting) leading to, or exacerbating, cutaneous AEs | 69% | Not typically seen in patients on MAPKi and other measures may be better for managing cutaneous AEs |
| *Eczematous Rash* |  |  |
| Only topical corticosteroids should be used in the management of MAPKi-induced eczematous rash, systemic corticosteroids are usually not indicated | 63% | Systemic corticosteroids or dupilumab may be used in severe cases |
| If an eczematous rash is present on the scalp, patients should consider using liquid or foam corticosteroids, or antifungal shampoo only if fungal infection is suspected | 66% | Disagreement regarding the use of antifungal shampoo only when infection is suspected |
| If pruritus is present with an eczematous rash, an antihistaminergic agent should be added, regardless of AE severity | 61% | Antihistamines may not be effective against itching and other skin-directed approaches may be more effective |
| *Acneiform Rash* | | |
| For mild acneiform rashes, patients should use a topical antibiotic | 63% | Other interventions may be preferred |
| For patients with severe acneiform rashes aged <8 years, the use of topical antibiotics is preferred | 49% | Severe acneiform rashes are rare in this age group, but oral antibiotics may be necessary |
| *Paronychia* | | |
| Topical beta blockers (e.g., timolol) should be considered for management of MAPKi-induced paronychia either (a) as a front-line alternative to antimicrobial therapy or (b) for antimicrobial-refractory paronychia | 38% | Topical beta blockers are not an established treatment for paronychia |
| For moderate paronychia, patients should use a high-potency topical corticosteroid with or without a systemic antibiotic | 54% | A combined topical therapy of a corticosteroid and antibiotic may be more commonly used |
| A systemic antibiotic is recommended if paronychia is not resolved with topical antibiotics | 73% | Only applies in cases of infection; other interventions may be considered |
| In the event of pus being present at the affected paronychia site, a collection of a sample from the affected site should be obtained, and systemic antibiotics initiated (tailored to the bacteria identified). Oral antibiotics should be used in combination with a topical corticosteroid | 63% | Topical antibiotics may be sufficient in some cases |
| *Gastrointestinal AEs* | | |
| Gastrointestinal AEs have been commonly reported with most MAPKi therapies | 56% | Some gastrointestinal AEs are drug specific, may be mild, and under reported |
| To prevent stomatitis, patients should be encouraged to follow an oral healthcare regimen including brushing teeth twice daily, brushing the tongue, and replacing toothbrushes at least every 3 months | 62% | Standard recommendations for oral care in patients receiving systemic cancer therapy can be applied |
| The patient and clinician should regularly inspect the mouth for stomatitis | 67% | Limited observations of stomatitis in these patients |
| Treatment with an analgesic or topical nystatin to be recommended for early-stage stomatitis | 52% | Nystatin should be used only if fungal infection is present; mouth washes may be an effective early treatment |
| To help manage pain as a result of stomatitis, oral mucositis gels could be recommended | 52% | Limited evidence to support use and oral analgesics are not specified in statement |
| To manage nausea, diarrhea, and constipation, patients should avoid fried, fatty, and high-salt foods, in consultation with a dietitian if possible | 44% | A well-balanced diet should be followed; dietitian consultation may not be required |
| If patients cannot manage nausea or vomiting with dietary changes alone, antiemetics may be used if clinically appropriate | 63% | Not considered as problematic AEs in these patients |
| If patients require treatment for nausea or vomiting due to gastritis, proton pump inhibitors should be used if appropriate (possible drug interactions should be checked by the clinician) | 63% | Limited experience of these AEs; proton pump inhibitors may not be suitable in these patients |
| *Ophthalmologic AEs* | | |
| MAPKi-related ocular events, including MAPKi-related glaucoma, BRAFi-related uveitis, and MEKi-related retinal events, are much rarer in pediatric patients than in adult patients | 63% | Ophthalmologic AEs are considered rare in all patients |
| An awareness of the serious but rare ocular AEs in pediatric patients receiving MAPKi therapies is required, including MAPKi‑related glaucoma and BRAFi-related uveitis | 69% | Awareness may be challenging as toxicities not observed by some clinicians treating these patients |
| In pediatric patients, the ocular AEs associated with MEK inhibitors include RPED, CSR, and outer retinal separation, although these are generally benign and self-limited | 52% | These AEs are observed very rarely and are not well understood |
| RPED, CSR, and outer retinal separation are sometimes asymptomatic and can be detected only with OCT, a specialized technique for imaging the optic nerve and retina | 46% | Understanding of these AEs and the need to screen for them is evolving |
| Referral to an ophthalmologist with experience managing patients treated with MAPKi therapy, if available, is encouraged for baseline exam prior to MAPKi initiation; a follow-up evaluation, including a dilated fundus exam and OCT, is recommended if there is a new visual complaint | 70% | Routine monitoring may be preferred, regardless of whether there are new visual symptoms; OCT may be limited to symptomatic patients |
| Ideally, a full ophthalmologic evaluation, including a dilated fundus exam, should be performed prior to MAPKi initiation to establish a baseline for future evaluations. OCT of the optic nerve and retina can be performed but is not mandatory | 59% | OCT should be performed at baseline in all children, where feasible |
| OCT is difficult to obtain in young pediatric patients, as it requires cooperation for several minutes to hold still and sit in a chair and therefore should only be considered if deemed necessary | 33% | OCT is feasible in most children aged 3 years and older |
| Sedated OCTs are not considered standard of care for young patients, and not all centers have the equipment needed for this | 61% | Sedation may not be necessary to achieve OCT in a young patient |
| A high frequency of ophthalmologic assessments is unnecessary and burdensome, given the low rate and severity of these AEs in pediatric patients. Thus, routine ophthalmic follow-up examinations may not be needed for all patients | 18% | Some AEs can be asymptomatic but serious so regular follow up is needed |
| If patients are diagnosed with RPED or CSR, regardless of whether visual acuity is affected, MAPKi therapy should be discontinued until the RPED or CSR is resolved | 57% | These AEs are rare and not well understood, as such approaches to management vary |
| In the case of retinal vein occlusion, MEKi should be permanently discontinued | 66% | Limited experience of this AE |
| *Cardiac AEs* |  |  |
| Cardiac AEs appear to be uncommon, although LVSD, pericardial effusion, and decreased LVEF have been commonly reported with some MEK inhibitors | 60% | Cardiac AEs are very rare in pediatric patients, with different MAPKi therapies resulting in different AEs |
| LVSD is the most commonly observed cardiac AE in pediatric patients treated with MEKi, and may present as asymptomatic decreases in ejection fraction or, in more severe cases, heart failure | 52% | Heart failure in these patients is extremely rare |
| Decreased LVEF is one of most commonly observed acute cardiac AE with MEKi, but is typically mild and self-limiting | 52% | Decreased LVEF is very rare in pediatric patients, but may result in treatment discontinuation |
| Patients with a history of MEKi therapy, especially those with a history of acute treatment-related changes in cardiac function, are at higher risk for cardiac AEs and more frequent monitoring by echocardiogram should be considered | 52% | There is limited data and lack of personal experience amongst the survey contributors to support this |
| Although cardiac AEs are infrequent, patients should be monitored with echocardiograms at the start of treatment, and then as clinically indicated | 50% | Regular monitoring with echocardiograms should be conducted in all patients on MAPKi therapies, not just in those with symptoms |
| For the first year of treatment, it is recommended to repeat echocardiograms every 3 months and less frequently thereafter in the absence of symptoms | 41% | There was no consensus on the frequency of monitoring, which ranged from monthly to yearly |
| If patients have other cardiovascular risk factors, consider monitoring with echocardiograms more frequently | 60% | There was no consensus on the frequency of monitoring |
| In pediatric patients who develop asymptomatic left ventricular dysfunction (e.g., a decline in LVEF of >10% from baseline), reducing the dose of MAPKi therapies and intensifying cardiac monitoring should be considered | 44% | Temporary treatment interruption or treatment discontinuation may be needed; input from a cardiologist is advised |
| LVEF reduction often resolves spontaneously, so if it is within a reasonable range for the patient's age and they are not symptomatic, no management approaches are needed (aside from regular echocardiography) | 43% | Dose reduction or treatment interruption may still be considered, in collaboration with a cardiologist |
| For symptomatic LVSD, or significant declines in LVEF (e.g., to <40%), MAPKi therapy should be interrupted | 63% | Treatment discontinuation or dose reduction may also be considered |
| A pediatric cardiologist must be consulted before considering MAPKi therapy resumption at a reduced dose | 59% | Consulting a cardiologist is an option, but the oncologist may be able to manage the patient in less complex cases |
| If patients have discontinued MAPKi due to decreased LVEF, but LVEF recovers to the lower limit of normal (per local institution) within 3 months, MAPKi therapy may be restarted at a reduced dose | 48% | Input from a cardiologist is required; restarting MAPKi therapy at a reduced dose should be based on clinical need |
| *Endocrine-Related AEs* |  |  |
| Decreased growth velocity has been reported with a type II RAFi | 62% | Lack of experience with type II RAFi among contributors |
| Decreased growth velocity may not be related to premature closure of growth plates; therefore, dependent on pubertal stage, decreased growth velocity may be reversible when treatment is discontinued | 55% | Long-term data is needed to determine if the effects on growth velocity are reversible |
| Weight gain is more common with MEK inhibitors compared with BRAFi/RAFi | 39% | No clear difference in the impact of MEKi and BRAFi/RAFi on weight gain |
| Weight gain is more pronounced with re-treatment or second MAPKi regimens | 5% | This is reported as being patient dependent or not observed |
| Weight gain with BRAFi is more commonly seen in adolescents than younger pediatric patients | 28% | Whether age is a factor in weight gain with BRAFi is unclear |
| Pediatric patients with AVP deficiency (formerly known as diabetes insipidus) who are on desmopressin therapy are at increased risk of developing hyponatremia when treated with MAPKi therapies | 32% | Limited experience with this clinical scenario |
| Fatty liver disease increases the risk of liver AEs during treatment with MAPKi therapies | 35% | Limited experience of liver risk |
| Patients with a suprasellar tumor and hypothalamic-pituitary dysfunction are at higher risk for endocrine AEs | 49% | Treatment-related endocrine AEs may be more evident in these patients |
| If appropriate, patients on MAPKi should be seen by an endocrinologist | 49% | Endocrinologists should be involved when there is clinical concern or abnormality |
| Pubertal status, bone age, and growth velocity should be continuously evaluated in all non-adult patients receiving MAPKi therapy | 59% | Bone age assessment is only required if there is a concern of delayed puberty or growth velocity |
| It is recommended to monitor height, weight, sitting height, and Tanner stage every 3 months | 49% | Sitting height not routinely measured and monitoring of these factors is generally done less frequently |
| In case of growth arrest, the dose and/or frequency of the MAPKi should be altered if appropriate | 30% | The approach depends on the specific patient and clinical scenario |
| The management of patients with AVP deficiency with MEKi should be initiated in consultation with a pediatric endocrinologist | 60% | Parents or the neuro-oncologist may manage dose adjustments |
| Pediatric patients with AVP deficiency receiving MAPKi therapy should have serum sodium levels monitored closely, especially during the initiation of therapy and after any dose adjustments (especially with trametinib) | 57% | Parents may adjust the medication based on clinical symptoms; unclear if specific data available on trametinib |
| To minimize the risk of hyponatremia in pediatric patients with AVP deficiency receiving MAPKi therapy, desmopressin dosing should be carefully adjusted based on fluid intake, urine output, and serum sodium levels | 56% | Often managed at home, but support from an endocrinologist is recommended |
| In cases of severe weight gain (>20% from baseline), consider discontinuation of MEK inhibitors if appropriate | 37% | An individualized approach is needed, with potential input from an endocrinologist and a nutritionist |
| *Laboratory Abnormalities* | | |
| More frequent laboratory screening is recommended for patients treated with type II RAFi | 26% | Unclear what type RAFi are being compared with; frequency of screening has not been established |
| Clinicians should aim to measure CPK at rest to ensure it is not elevated due to other muscle injury or vigorous exercise | 66% | Challenging to achieve in young patients and CPK elevation is commonly observed |
| To monitor for CPK elevation, clinicians should perform blood tests every 1–3 months at the start of treatment, later transitioning to tests every 4–6 months | 62% | Frequency of monitoring not established |
| If patients are diagnosed with rhabdomyolysis, permanent MAPKi discontinuation is recommended | 57% | Dose reduction and/or treatment interruption may be considered |
| *Other AEs* | | |
| Intra-tumoral hemorrhage is a recognized AE of MAPKi therapies | 39% | A rare AE, where the cause is unknown and the relationship with different MAPKi drug classes has not been determined |
| Fatigue is underreported as a dose-modifying or significant AE and is more pronounced with re-treatment or second MAPKi regimens | 35% | Fatigue is an established AE with MAPKi but whether there is any correlation with line of therapy is unclear |
| Fatigue as a result of MAPKi can have a significant impact on patient QoL | 66% | Limited experience of fatigue impacting patient quality of life |
| Fever is more commonly observed with combination therapy compared with BRAFi/MEKi monotherapy | 30% | Lack of consensus on whether combination therapy is more likely to result in fever than monotherapy |
| It is unknown whether MAPKi therapies may be associated with cognitive effects, or whether the degree of brain penetrance of the MAPKi may impact this risk | 54% | Limited data available on this topic |
| *Pyrexia* | | |
| For the first occurrence of moderate fever (>39˚C), MAPKi therapy should be discontinued until fever subsides | 44% | Stopping MAPKi may not be necessary and may depend on the drug or combination being used |
| In cases of pyrexia syndrome (>3 days of fever in 30 days), corticosteroid prophylaxis is recommended | 22% | Corticosteroid use is not the preferred treatment approach |
| In cases of recurrent pyrexia syndrome (>3 days of fever in 30 days), antipyretic prophylaxis is recommended rather than corticosteroids | 29% | Addition of another medication to treat pyrexia is considered on a patient-specific basis |

AE = adverse event; AVP = arginine vasopressin; BRAFi = BRAF inhibitor; CPK = creatine phosphokinase; CTCAE = Common Terminology Criteria for Adverse Events; CSR = central serous retinopathy; LVEF = left ventricular ejection fraction; LVSD = left ventricular systolic dysfunction; MAPKi = MAPK inhibitor; MEKi = MEK inhibitor; OCT = optical coherence tomography; QoL = quality of life; RAFi = RAF inhibitor; RPED = retinal pigment epithelial defects.
